# What Data to Use for Forest Conservation Planning? A Comparison of Coarse Open and Detailed Proprietary Forest Inventory Data in Finland

**DOI:** 10.1371/journal.pone.0135926

**Published:** 2015-08-28

**Authors:** Joona Lehtomäki, Sakari Tuominen, Tuuli Toivonen, Antti Leinonen

**Affiliations:** 1 Department of Biosciences, University of Helsinki, Helsinki, Finland; 2 Finnish Environment Institute, Natural Environment Centre, Helsinki, Finland; 3 Natural Resources Institute Finland, Vantaa, Finland; 4 Finnish Forest Centre (Suomen Metsäkeskus), Kajaani, Finland; University of Brasilia, BRAZIL

## Abstract

The boreal region is facing intensifying resource extraction pressure, but the lack of comprehensive biodiversity data makes operative forest conservation planning difficult. Many countries have implemented forest inventory schemes and are making extensive and up-to-date forest databases increasingly available. Some of the more detailed inventory databases, however, remain proprietary and unavailable for conservation planning. Here, we investigate how well different open and proprietary forest inventory data sets suit the purpose of conservation prioritization in Finland. We also explore how much priorities are affected by using the less accurate but open data. First, we construct a set of indices for forest conservation value based on quantitative information commonly found in forest inventories. These include the maturity of the trees, tree species composition, and site fertility. Secondly, using these data and accounting for connectivity between forest types, we investigate the patterns in conservation priority. For prioritization, we use Zonation, a method and software for spatial conservation prioritization. We then validate the prioritizations by comparing them to known areas of high conservation value. We show that the overall priority patterns are relatively consistent across different data sources and analysis options. However, the coarse data cannot be used to accurately identify the high-priority areas as it misses much of the fine-scale variation in forest structures. We conclude that, while inventory data collected for forestry purposes may be useful for forest conservation purposes, it needs to be detailed enough to be able to account for more fine-scaled features of high conservation value. These results underline the importance of making detailed inventory data publicly available. Finally, we discuss how the prioritization methodology we used could be integrated into operative forest management, especially in countries in the boreal zone.

## Introduction

### Informative conservation decision-making depends on available data

Biodiversity conservation deals with multifaceted and complex problems [1] that call for inter- or transdisciplinary research and decision-making [1–3]. Several different kinds of data are typically required [4], such as spatial data on species distributions, habitats and ecosystem services [5,6], costs associated with conservation actions [7], the structure and representativeness of the existing reserve network [8], and increasingly information about the present and future state of dynamic environments [9,10] and anthropogenic threats [11,12]. Furthermore, on-ground conservation decisions are almost always tied to a relatively fine spatial scale which implies that the data used for conservation prioritization should also have resolution relevant for the prioritization problem at hand [6,13]. Spatial conservation prioritization [14,15] is a form of conservation assessment primarily interested in when, where, and how should conservation action be taken in order to achieve conservation goals [16,17]. It can be embedded within a broader context of conservation planning [18] that can be described as a complete operational model covering all the stages needed for successful conservation action including assessment, planning and management. Conservation prioritization problems have been extensively studied conceptually and mathematically for many years [19] and consequently many software methods for solving a wide array of problems have also been published [20–24].

Most of the contemporary approaches on spatial conservation prioritization are based on the concept of complementarity, which can be defined as a property of a prioritization solution whereby high-priority sites complement each other in terms of biodiversity features they contain. In other words, sites work together efficiently in achieving conservation objectives [25]. If a site has unique biodiversity features such as species, it is often considered irreplaceable and sites with high irreplaceability are typically considered high conservation priorities [15]. Connectivity is another central concept for spatial conservation prioritization and planning [26]. The term connectivity is commonly used to refer to measures of spatial connectedness of a network of sites connected by species’ dispersal with higher connectivity usually implying increased species persistence [27]. In practice, however, operationalizing connectivity in spatial conservation prioritization has been difficult because of multitude of definitions [28] and computational intractability [29]. Nevertheless, enhancing connectivity is often promoted as an important conservation strategy [28,30,31], although it may come at the expense of high-quality sites that are poorly connected [13,26]. Irrespective of the spatial conservation prioritization method employed, validating the results is an important, but often overlooked part of the whole prioritization. Maps and other results of prioritization assessments are often produced assuming that the input data is of sensible quality and thus the priorities reflect on-ground reality adequately [32], but this assumption needs to be validated against independent validation data. Creating informative and accurate conservation prioritization results therefore hinges on the availability of reliable input and validation data.

A number of studies have been published regarding the pattern of distribution of biodiversity and new technological advancements, such as relatively cheap and very accurate remote sensors, have led to an increase in the available biodiversity data [33]. However, in most regions of the world the primary biodiversity data for conservation decision-making still remains scarce [34,35] and are often biased in terms of species representation, areas sampled and spatiotemporal accuracy [36,37]. For most species, and most parts of the world, we simply do not have sufficient data [35,38] and even when we do, it is not necessarily accessible. Sharing data is especially desirable from the decision-making point of view because of the many benefits it entails, such as enabling integrative and synthesizing science [39], enabling exploration of new topics not envisioned by the data originators [40], and providing more verifiable research for policymakers [41]. Many public and private organizations collect and maintain research and monitoring databases that could be valuable for conservation decision-making, but remain unavailable because of political or technical barriers for data sharing. There are good reasons for withholding the data, such as detailed location data on endangered species or confidential information concerning the privacy of individuals [40,42,43]. Restricting access to such information is not only an ethical obligation, but also often a legal one. Thus, the availability of potentially useful biodiversity data remains restricted, despite the fact that conservation decisions still have to be made [35,44]. With great potential for better informed decision-making, open access to relevant data is crucial for addressing the increasingly complex conservation issues the world is facing [12,34,43,45,46].

### Forest inventory data for spatial conservation prioritization in the boreal zone

The circumpolar boreal forest is the second largest biome in the world [47]. Countries in the boreal zone have traditionally utilized their forest-based natural resources extensively, which has led to changes in forest structure, species composition, habitat diversity, and large-scale disturbance dynamics [48–52]. While it is not the most species-rich or threatened biome on the planet [53,54], there are still many reasons for increasing conservation efforts in the boreal zone. First, boreal forests host a great number of highly specialized species that are dependent on resources such as dead wood [55–57]. Many of these species have become endangered because of intensive forestry practices. Second, because of their large extent and biomass, boreal forests have a major role in carbon sequestration and climate change adaption [47,58]. Third, many parts of boreal zone, especially in the Russian Federation and Canada, remain inaccessible presenting an opportunity to protect large tracts of relatively intact forest [59,60].

In boreal zone and elsewhere, effective conservation planning should ideally be done simultaneously with general land-use and natural resource use planning [6]. This further emphasizes the need to be able to synthesize and utilize data from various sources. Data for land use and natural resources use planning may also be useful for conservation planning, assuming that they act as surrogates for biodiversity features of conservation interest. The upside is that resources allocated for collecting these types of data usually exceed those allocated for conservation-related data collection. For example, countries with an active forest sector typically have high-resolution, national, forest inventory systems (NFIs) in place [61,62]. In addition to NFIs, many other public and private operators collect detailed forest inventory data for their own operational planning often at the national or regional scale. Recently, governments and public research institutions in particular have started opening up their databases. For example, the Finnish Forest Research Institute has opened up their multi-source national forest inventory database (http://www.metla.fi/ohjelma/vmi/vmi-moni-en.htm).

Forest inventory data has historically been collected to assess the productive functions of forests [61], but broad-scale NFIs are increasingly being used for monitoring forest biodiversity particularly in boreal forests [63]. Following the classification by Corona et al. [63], biodiversity indicators estimated from forest inventory data can be classified into two categories: (i) compositional indicators directly measuring biodiversity, and (ii) structural indicators based on key structural features (e.g. variability in tree size and the amount of dead wood) acting as correlates or surrogates for biodiversity [63]. The latter approach largely relies on the assumption that high structural and tree species diversity provides more habitats for different forest species [63–65]. It also requires that the features can be reliably estimated from forest inventory data [66,67]. The approach based on structural indicators also has many desirable qualities from the perspective of spatial conservation prioritization. First, as structural components are comparatively easy to measure, data about them are commonly included in forest inventories [61,63]. Second, the effects different forest management options have on structural features and thus on biodiversity can be easily assessed [68], enabling comparisons between different management scenarios. Third, forest inventories are typically repeated periodically, making it possible to monitor changing conditions [63]. Fourth, since forest inventory data still are primarily collected for forest management and planning purposes, conservation prioritization based on forest inventory data can be more easily understood by forestry practitioners. Finally, the data, and thus the results of conservation prioritization analyses, are produced at a resolution directly relevant for operative planning. However, validating the results is even more important when relying on surrogate data such as structural forest inventory data. At the structural level, one can use the locations of known conservation value, such as existing protected areas, as crude validation data which should, on average, be more valuable than the surrounding (commercially managed) landscape.

### Aims and scope

Here, we develop a set of conservation prioritization analyses based on freely available and proprietary forest inventory data with a varying degree of detail across the province of South Savonia, Finland. We use the conservation prioritization software Zonation to develop complementarity-based priority maps. Accounting for connectivity in spatial conservation prioritization can be ecologically justified [25] and often called for in conservation implementation [69]. Therefore, we also include connectivity considerations in our Zonation analyses. With this approach, we are studying the following questions:

1. Can conservation prioritization analysis based on forest inventory data capture conservation value in boreal managed forest landscapes?
2. How well does freely available coarse forest inventory data perform compared to more detailed proprietary stand-based inventory data?

Furthermore, given the differences in the data reliability and prioritization results, we discuss under what kind of planning circumstances is open but coarse inventory data sufficient for informative conservation decision-making?

We limit our attention to the effects that different data sources have on the quality of spatial prioritization, and we acknowledge that the computational analysis described here is just one part of a full conservation planning process [20,70]. While the results will be case-specific to a certain degree, the procedure itself is applicable to other countries that have similar forest inventory data available. The results should be applicable to countries with a similar forest management history and current forest and conservation management needs.

To encourage other scientists and practitioners to build upon the work presented here, we also make available the full analysis implementation (see S1 Appendix) and the code necessary to produce the results from the prioritization analyses. While the proprietary data we have used cannot be shared because of privacy issues, we have made all the stages of the implementation openly available for examination and re-use.

## Material and Methods

### Study area

The study area covers the region of Southern Savonia located in southeastern part of Finland. South Savonia is one of 13 regional administrative units of the Finnish Forest Centre (FFC). The region is ca. 13990 km^2^ and characterized by a large number of lakes and fragmented waterways, which cover ca. 25% of the total area. Of the land area, approximately 88% is forestry land that can further be divided into mineral soils (79%) and mires (21%). Together, forests on mineral soils and mires form a gradually varying landscape mosaic in the study region. The south boreal vegetation zone covers the whole region and forests are mostly dominated by the Scots pine (*Pinus sylvestris*) and the Norway spruce (*Picea abies*), mixed with varying amounts of broadleaved trees. Land ownership is highly fragmented, with private forest owners being the largest group (77.3%) followed by private companies (11.5%) and the state (6.2%) [71]. Most of the forestry land is under silvicultural management and only 2.5% is strictly protected, which is the same as the average for forestry land in southern Finland. Whereas private forest land has several operators working on it (including the FFC), the state-owned land is managed by a single organization, Metsähallitus (the Finnish Forest and Park Service, which is further divided into two independent departments: the Forestry Department manages the Finnish state production forests and the Natural Heritage Services (NHS) manages forests outside of commercial operations, including protected areas.

### Study design

**Fig 1:**
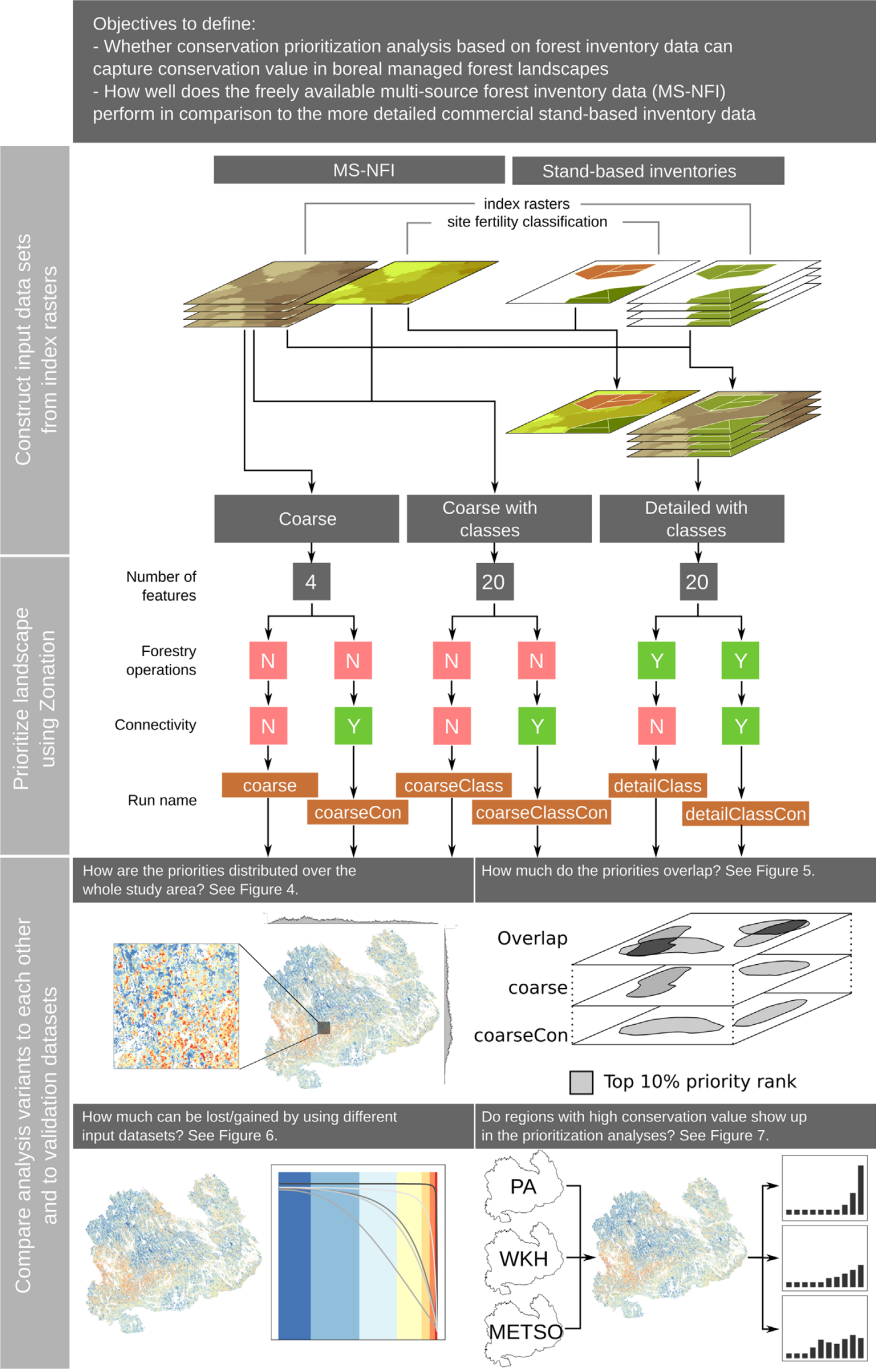
Schematic of the flow of analysis. The figure is divided into three sections. The first describes how we combined the index rasters to produce the input data sets used in the prioritization analyses. The second summarizes the main characteristics of spatial conservation prioritization analyses done using Zonation. The third section summarizes how we analyzed the priority rank maps produced by the six different Zonation analyses. PA = protected areas, WKH = woodland key-habitats, METSO = protected areas from the METSO programme. Rank priority maps printed under a CC BY license, with permission from original copyright holder the Finnish Forest Contre, 2015.

Fig 1 presents the design of our study and Table 1 the data sets used in the analysis. To address the main objectives, we 1) acquired coarse and detailed forestry inventory data from Southern Savonia, 2) calculated comparable surrogate indices of conservation value out of these data, 3) carried out six different conservation prioritizations using three different input data sets and testing the influence of connectivity transformations, and 4) compared all prioritization results to each other and areas with known high conservation value. We included both forests on mineral soils and mires in the analyses, because ecologically the forests and mires are often linked by partly overlapping species pools and connecivity. However, the surrogate indices reflect only the conservation value of forests on and thus the results are uninformative for mires.

**Table 1:** The spatial data sets used in the study. Column Use indicates whether the data set is used as input for the analyses or in validation.

Data set	Type	Use	Coverage (%)	Mean site size (ha)	Source^a^	Availability
Multi-source National Forest Inventory Data	Raster	Analysis	100.00	NA	FRI	Freely available
Stand-based forest inventory data	Vector	Analysis	43.94	NA	FFC	Available for research, strict conditions
Stand-based forest inventory data	Vector	Analysis	2.34	NA	NHS	Available for research, lax conditions
Protected areas	Vector	Validation	1.87	13.54	FPS	Available for research, lax conditions
Woodland key-habitats	Vector	Validation	0.50	0.61	FC	Available for research, strict conditions
METSO-deals	Vector	Validation	0.13	5.36	CEDTE	Available for research, lax conditions

^a^ FRI = Finnish Forest Research Institute, FFC = Finnish Forest Centre, NHS = Metsähallitus (Finnish Forest and Park Service) Natural Heritage Services, CEDTE = Centre for Economic Development, Transport and the Environment

### Data sets

#### Coarse data

**Fig 2:**
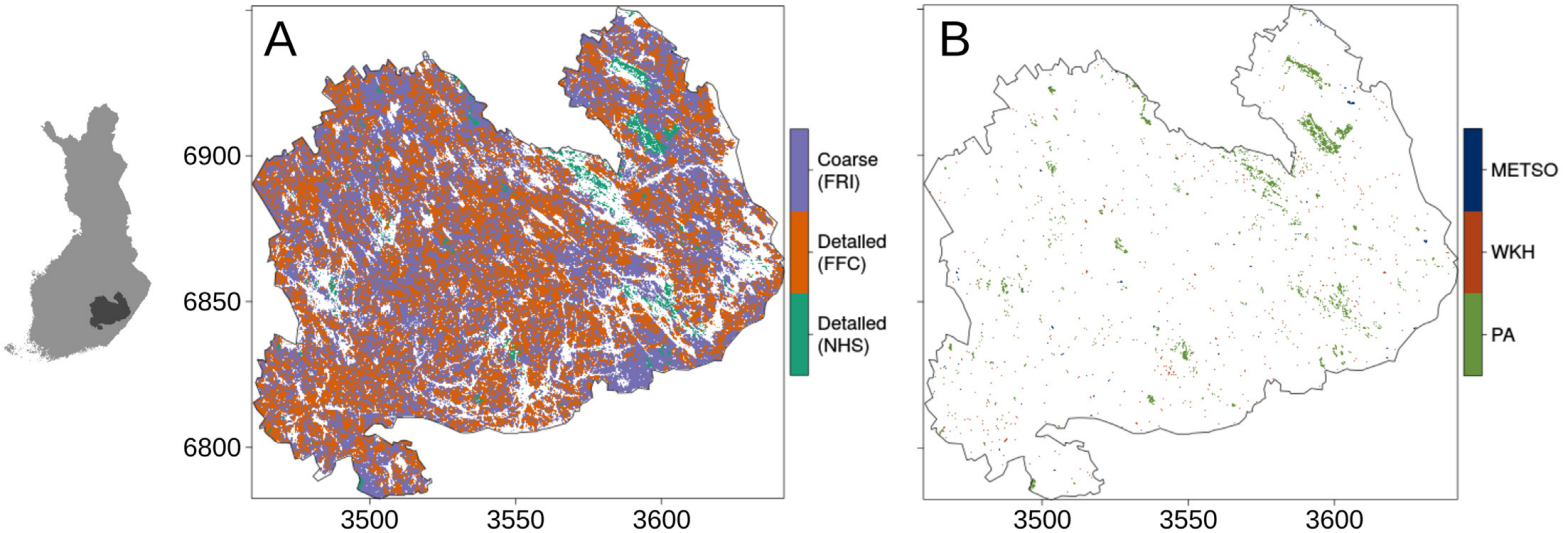
The spatial coverage of data sets. (A) Regions of the study area covered by the different forest inventory data. White regions within the study area are either water bodies or more densely populated areas. (B) Regions of the study area covered by the different validation data sets. Labels on the x- and y-axes correspond to the kilometer component of the Finland Uniform Coordinate System (EPSG 2393), i.e. the distance between ticks is equal to 50 kilometers. Maps on detailed data from the FFC and WKH printed under a CC BY license, with permission from original copyright holder the Finnish Forest Centre, 2015.

The coarse data used in this study were based on the multi-source national forest inventory (MS-NFI) developed and maintained by the Finnish Forest Research Institute (FRI). The MS-NFI method employs satellite images, digital maps and field measurements to estimate thematic digital maps about structural features of the forest across Finland at a spatial resolution of 20 m. MS-NFI data collection covers all land-use classes and ownership categories throughout the country [62,72,73] including the study area (Table 1 and Fig 2). The final data product contains over 40 forest variables in the form of thematic maps, including the volumes by tree species and timber assortments, stand mean variables, the biomass by tree species groups and tree compartments and forest site type characteristics (e.g. [62,73,74]). In Finland, the MS-NFI is being used mostly for regional level forestry planning, but it has also been used for large-scale conservation prioritization studies [13,74,75]. The MS-NFI data has been publicly available since late 2012, the thematic maps can be viewed through a web portal and the rasters can be downloaded through a file service [76].

The conservation value indices used for the prioritization require that information on both the average diameter and the volume are available for each tree species group. The standard MS-NFI rasters include only one estimate for average diameter over all tree species groups. In order to calculate estimates of average diameter for each tree species group, stand level variables were derived from the MS-NFI by the way of automatic stand delineation. We did the stand delineation in the study area by using automatic segmentation (see also S1 Appendix) on the MS-NFI forest maps of the 10th iteration of the National Forest Inventory done between years 2004 and 2008. As the input data for the segmentation, we used the thematic map layers on stand mean height and volumes of the tree species groups: pine, spruce, birch and other broadleaved trees. The segmentation was carried out using a modified implementation of the “segmentation with directed trees” algorithm by Nagendra & Goldberg [77]. The algorithm is based on using the local edge gradient for linking individual pixels into larger spatially continuous units, i.e. segments. The automatic segmentation process is guided by parameters such as heterogeneity allowed within the segments and the desired minimum size of the segments [78]. The desired size of segments was approx. 1–2 ha. We calculated the stand level variables as average values of the individual pixels within each segment and the variables per tree species by weighting the pixel level variables by the volumes of individual tree species.

#### Detailed data

Here, we refer to detailed data for stands or forestry compartments. The data are produced by a combination of direct field inventories and a representative plot-based sampling system. Nowadays, inventory data are also updated using remote sensing data (LiDAR). These data are collated to provide very fine scale information for forest management planning [72] by different authorities and forestry organizations depending on land tenure. For this study, we used data from two authorities operating in the study region: the FFC on private land and Metsähallitus NHS on public land.

The FFC inventories forest stands only on need-basis or when forestry operations take place. Therefore, some of the inventory data can be relatively old and does not represent the current state of the forest very well. To account for this, we only used data gathered in year 2000 or after covering ~44% of the land area (Table 1 and Fig 2). In addition, we used spatial data on the planned forestry operations, such as thinnings and clear-cuts. We used these data to discount the conservation value of forest areas that are planned to go through forest operations of varying degree. The forest inventory data gathered and managed by the FFC are not freely available as the Finnish Personal Data Act restricts the distribution of the data at a resolution that allows linkage of the data to properties of individual forest owners. It is possible, however, to get access to the data for research purposes [79].

Metsähallitus NHS has a similar inventory system in place on public land. Their information system updates the database annually to simulate the growth of forests, and consequently no filtering was needed. We received detailed stand-based data from NHS after signing a research collaboration agreement. We were unable to get any data from regions governed by Metsähallitus Forestry. Detailed data from Metsähallitus NHS covers ~2.4% of the land area in Southern Savonia.

#### Data for validation

We used three different data sets for validating the prioritization results: spatial delineations of 1) the established protected area network, 2) woodland key-habitats, and 3) recently acquired protected areas (Table 1).

Metsähallitus NHS maintains the data on established protected areas and a spatial database is publically available. Protected areas also cover mires, but for validation, we used only protected areas on mineral soils (~1.9% of the whole landscape). Protected areas have often been established for reasons other than their high biodiversity value and this could be the case in Southern Savonia as well. However, many of the protected areas in the region—including two national parks—contain relatively old forests that have been outside commercial forestry management for decades and thus probably contain features important for forest biodiversity.

Woodland key-habitats (WKH) are a conservation instrument designed for maintaining landscape-level biodiversity in production forests by delineating and preserving small habitat patches of high conservation value [80]. The concept is in use in many Fennoscandian and Baltic countries and, while their effectiveness as a conservation measure varies depending on the country and definition [80–82], WKHs seem to be hotspots for dead wood dependent and red-listed species, and for species richness in general [80]. Because of potential privacy issues, the exact spatial locations of WKHs are not public information, but the data is available for research use.

Recently acquired protected areas are related to the forest biodiversity conservation programme METSO that is an ongoing effort to halt the decline of forest biodiversity by 2016 [69]. Individual forest owners can offer their forest property for protection, and if the particular offer fulfills given scientific selection criteria, it is admitted into the programme through either a permanent or temporary (10 years) conservation contract. The forest owner then receives a tax-free compensation based on the economic value of the growing stock and timber [83]. The sites selected in METSO are ecologically more valuable than average Finnish forests, containing more dead wood as well as many red-listed species [84]. We used only areas with permanent conservation contracts for validation, as the conservation effectiveness of temporary or fixed-term contracts is questionable [84]. These data are not publicly available before they become integrated into the main protected areas database, but it can be accessed for research use.

In terms of size, the mean size of distinct spatial units in the protected area data set is higher (13.54 ha, Table 1) than in the WKH data set (0.61 ha) or the recently acquired protected areas data set (5.36 ha).

### Calculating conservation value indices from original data

We reclassified the original forestry data (both coarse and detailed) into four tree species groups: pine, spruce, birch, or other broadleaved. We calculated an index of conservation value per pixel for each of the tree species groups in each of the input data sets separately (Fig 1). This index measures an expert-derived view on how the average diameter and the volume of the growing stock relate to ecological features desirable for conservation. We transformed the average diameter of the growing stock per tree species group by a sigmoidal benefit function (see S1 Appendix and S1 Fig) and then multiplied the transformed value by the volume of the growing stock. A similar approach has been used earlier in large-scale conservation prioritizations [13,74] and in species-oriented prioritization [75].

**Fig 3:**
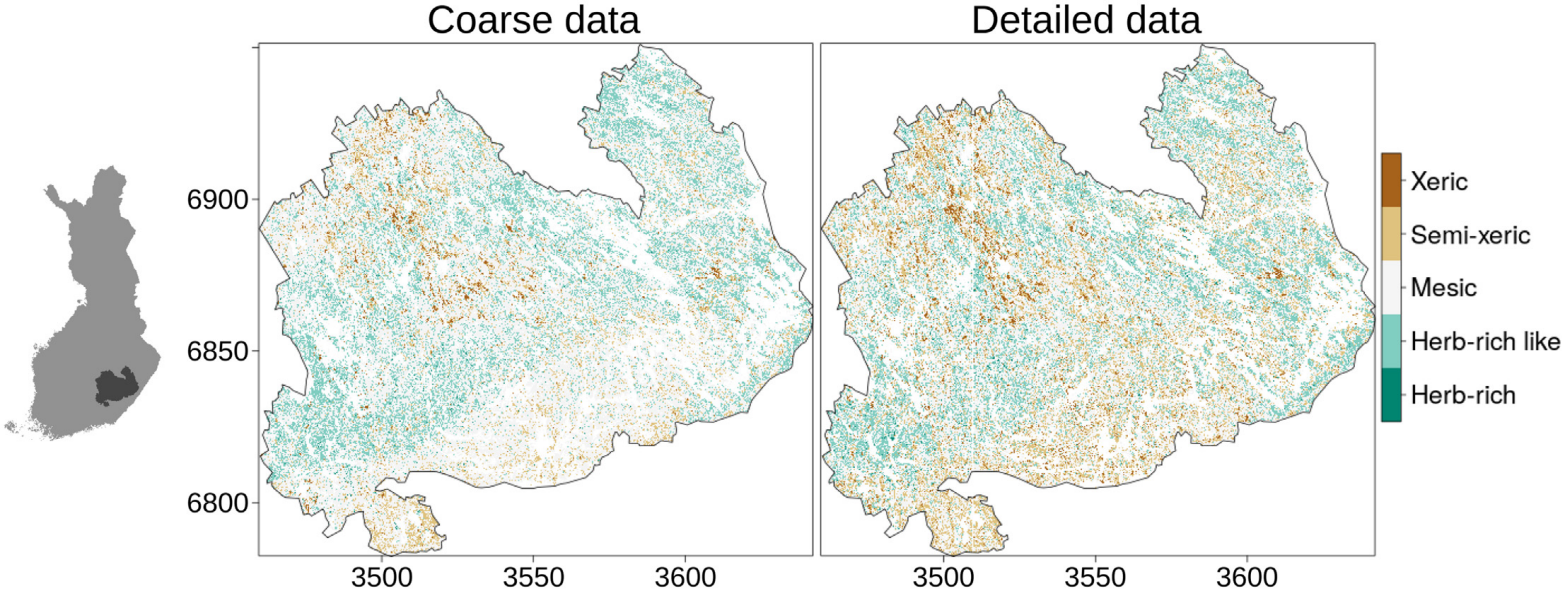
The spatial distribution of site fertility classes in the coarse and detailed data. Labels on the x- and y-axis correspond to the kilometer component of the Finland Uniform Coordinate System (EPSG 2393), i.e. the distance between ticks equals to 50 kilometers. Map on the detailed data printed under a CC BY license, with permission from original copyright holder the Finnish Forest Centre, 2015.

All available data sets had information on site fertility class, which is often also associated with the formation of specific forest microhabitats. We hypothesized that using a classification scheme would emphasize rarer forest types that typically have higher biodiversity value. Therefore we created two input data sets based on the coarse data: One with just the four index rasters (“Coarse”), one with the four index rasters each divided into five site fertility classes (“Coarse with classes”, Figs 1 and 3). The division into site fertility classes produces 20 tree species group—site fertility type classes, which we refer to as forest types. We did the same classification operation to the detailed data producing the input data set “Detailed with classes”. We also hypothesized that prioritization based on the more detailed—and more precise—inventory data would outperform those based on the coarser data. Note that the input data set “Detailed with classes” is actually a combination of coarse and detailed as the detailed data only covered ~46% of the landscape (Table 1). Both “Coarse” and “Coarse with classes” are completely based on MS-NFI and thus publicly available data.

We converted all detailed vector data to rasters of the same resolution (20 m) and extent as the MS-NFI. For computational reasons, we aggregated the data to 60 x 60 m pixel size using ArcGIS (version 10.2.1) [85]. We wanted to retain as high a resolution as possible because conservation prioritization analyses should be carried out at a spatial scale that is informative about ecological components (e.g. connectivity and average size of habitat patches) and relevant at the scale of operative planning [13]. For calculating conservation value indices at this resolution, we used custom-made geospatial scripts based on Python [86] (version 2.7.2) bindings to GDAL [87] (version 1.10.1).

### Prioritizing locations for conservation

For the spatial prioritization, we used Zonation [21,88] (version 4.0 [89]). It is a complementarity-based software that operates on a set of input rasters describing the occurrence levels of biodiversity features across the landscape; in our case the features were the index rasters of forest conservation value. Initially Zonation considers the values of all input rasters in each pixel in the landscape. It then proceeds by iteratively removing the least valuable cells simultaneously accounting for the occurrence of features in cells, the remaining occurrence of each feature across the landscape, and connectivity. At each iteration, the features are normalized by their remaining range-size, meaning that as a feature becomes rarer during the cell-removal process, its relative significance increases. The repeated range-size normalization [90] leads to maintenance of a balance between all features at all iterations. In the end, Zonation has heuristically ranked the whole landscape according to its conservation priority. If used for designing a protected area network, Zonation can account for complementarity and irreplaceability of high-priority sites. We encourage the reader to refer to a large body of existing literature for the conceptual background [88,90,91], the exact operational principles [20,89], and the different applications of Zonation (e.g. [74,75,92,93]).

Following best-practices for constructing Zonation runs [20], we started from the simplest possible configurations, enabling more complex features one at a time. This way, it is possible to test for the exact effects each component of the analysis introduces and the sensitivity of the results to different parameter values. After testing with several different combinations, we set up two runs for each input data sets (“Coarse”, “Coarse with classes”, and “Detailed with classes”): one with and one without connectivity. Thus, we completed six different analysis runs in total (“coarse”, “coarseCon”, “coarseClass”, “coarseClassCon”, “detailClass”, “detailClassCon” in Fig 1).

All six runs shared certain Zonation configuration options. We used the additive benefit function mode in Zonation [94], because it is appropriate when dealing with habitat data that acts as surrogate for biodiversity at large [74]. It is possible to weight input biodiversity features differently if there is a reason to do so. For example, the threat level of a species can be used for weighting, but any scheme reflecting the (often subjective) valuation of features can be used [20]. For the current study, we based the weighting scheme (S1 Table) on expert opinion so that more weight was given to forests with deciduous tree species on fertile soil. These types of forests are also emphasized in the METSO programme [69].

Runs also had differences (Fig 1). The number of biodiversity features (the index rasters) varied from four (“coarse” and “coarseCon”) to twenty (“coarseClass”, “coarseClassCon”, “detailClass” and “detailClassCon”). Runs based on the detailed data (“detailClass” and “detailClassCon”) used additional information about planned forestry operations. Technically, we implemented this in Zonation using the data as a condition layer, where local quality (as measured by the values in the index rasters) was reduced at locations with forestry operations [89]. We gave each forestry operation a value between 0.0 (all conservation value lost) and 1.0 (all conservation value retained) reflecting the subjective view on the effect of that operation. Runs “coarse”, “coarseClass”, and “detailClass” are do not include any connectivity transformations. Runs “coarseCon”, “coarseClassCon”, and “detailClassCon” on the other hand account for connectivity between different forest types (S2 and S3 Tables). We used the matrix-connectivity feature of Zonation [74,89], in which partially similar forest types facilitate connectivity for each other. While the landscape is divided into different forest types, each forest type still contribute to the connectivity of every other forest type more than non-forest habitats [89]. The spatial scale of the connectivity transformation effect in Zonation is controlled by a feature-specific parameter (α), which is derived from the scale of landscape use of each species or community occupying a habitat type [13,20,74]. We used a value of α (0.001), which corresponds to an average dispersal distance of 2.0 kilometers in a negative exponential dispersal kernel. See Lehtomäki et al. [74] and Sirkiä et al. [75] for further discussion and references about the distances chosen. We also tested the sensitivity of results, replicating the analysis with scales of 0.2 and 4.0 km, but these did not change the qualitative interpretation of results significantly. See Arponen et al. [13] for further discussion on the role of the spatial scale.

### Comparison and validation of analysis runs

One of main outputs of a Zonation analysis is a raster file, so called rank raster, representing the ranking of the landscape in terms of conservation priority. Low values (minimum of 0) indicate low conservation priority and high values (maximum of 1) high conservation priority. We examined the spatial patterns of the resulting rank rasters at different spatial scales. We did comparisons between (i) runs based on the same input data set but different analysis settings (i.e. the effect of connectivity) and (ii) between different input data sets analyzed using the same settings (i.e. the effect of the input data). We performed all comparisons using the standard Zonation outputs and data. We used R (version 3.1.0) statistical language [95] and the zonator R-package [96] (version 0.3.10).

Visual examination of the rank rasters should give an initial idea how well the different runs—and hence the different input data sets—converge especially in terms of high and low conservation priorities. We also compared the spatial overlap between analyses by calculating Jaccard coefficients (the intersection of two sets divided by the union of those sets) for different priority intervals. In other words, we divided each rank raster into 10 equal intervals and compared each interval of each rank raster to each interval of every other rank raster. This way we could compare, for example, the spatial overlap of the best 10% of the landscape in runs “coarse” and “coarseClass”, but also the worst 10% in “coarse” with the best 10% in “coarseClass”.

We also examined how well different prioritization runs were able to identify forest regions with known high conservation value. We did this by overlaying the priority distributions within areas covered by each of the validation data sets.

Using Zonation, it is possible to load the rank order of the solution (i.e. the inverse removal order of the cells) while using different input features or Zonation options. This procedure allows the examination of how much performance is lost when the analysis criteria and evaluation criteria differ [20,89]. For each analysis run, Zonation produces information on how large a fraction of the distributions of each biodiversity feature can be represented by a particular fraction (e.g. top 10%) of the landscape. We evaluated how much these fractions, so called representation levels, differ when the input features are based on the detailed data (“detailClass”) but the priority ranking is taken from analyses based on the coarser data (“coarse” and “coarseClass”). Assuming that the detailed data is also more correct, we can then answer the question of how much feature representation we risk losing if relying only on the coarser data.

## Results

### Spatial patterns in the rank rasters

**Fig 4:**
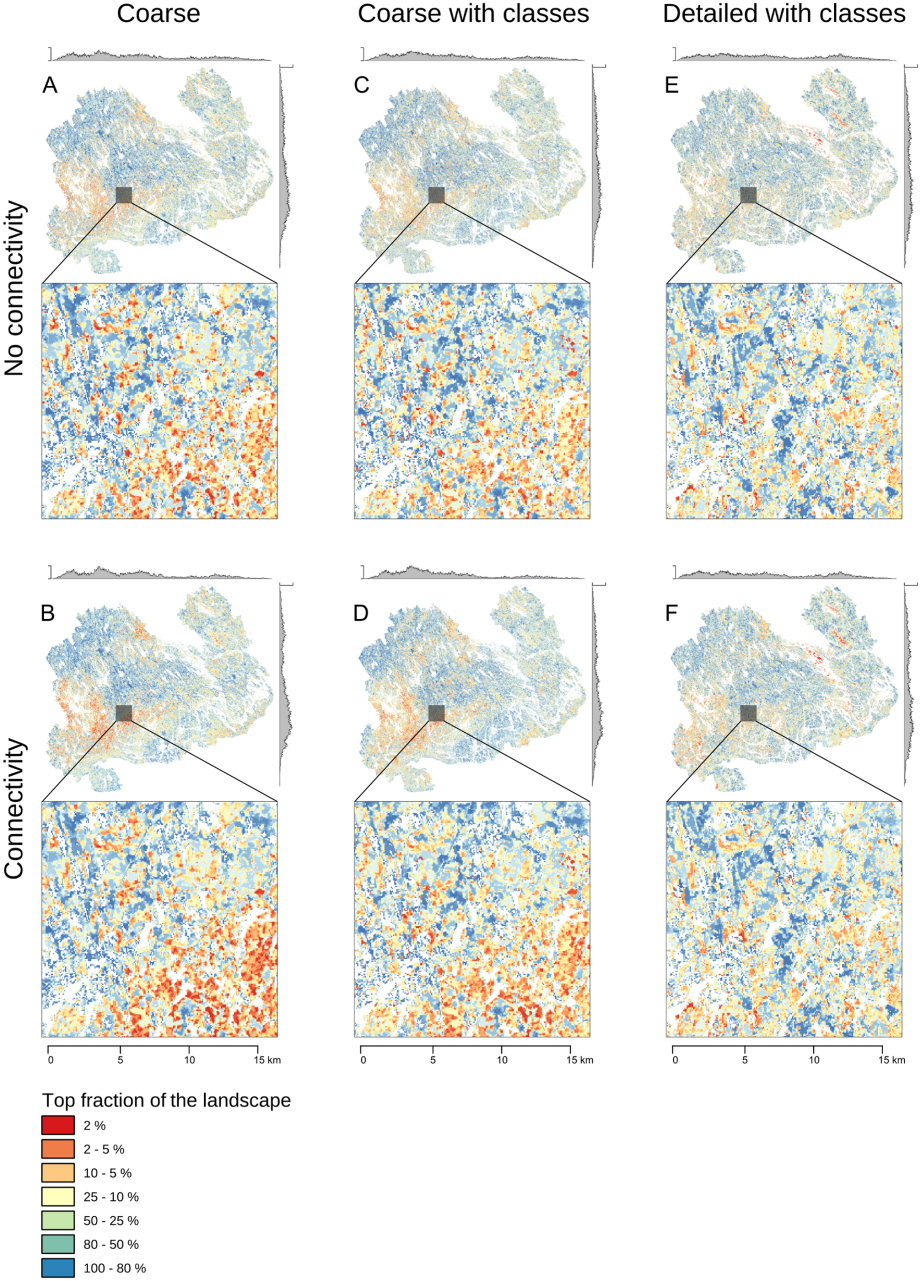
Priority rank maps for runs “coarse” (A), “coarseCon” (B), “coarseClass” (C), “coarseClassCon” (D), “detailClass” (E), and “detailClassCon” (F). The marginal plots on top and on the left side of each panel show the count of cells in the top 10% of the landscape (with highest priority) along both latitudinal and longitudinal gradients. The y-axis scale is the same for marginal plots ranging from 0 to 150. The insets expand the priority pattern from a selected smaller area. Rank priority maps D and E printed under a CC BY license, with permission from original copyright holder the Finnish Forest Centre, 2015.

Overall, the spatial patterns in the rank rasters were roughly consistent across runs not accounting for connectivity (Fig 4A, 4C and 4E). A major concentration of high-priority areas was identified in the southwestern corner of the study area. Classifying the coarse input data set according to the site fertility classification (“coarseClass”, see Fig 1) had only a minor effect of distributing the high-priority areas more equally across the study area (Fig 4A and 4C). Zonation tries to retain a balanced representation of all features throughout the analysis and therefore introducing more classes (i.e. features) will produce more even distribution of high-priority areas unless the most valuable features are spatially aggregated. “detailClass”, which is based on the more detailed data, produced a different priority pattern (Fig 4E). High-priority areas are distributed even more equally over the study area (marginal plots in Fig 4E). Regions of concentrated of high-priority areas also partially shift towards the northeastern part of the study area. This shift is at least partly explained by the fact that the more detailed data gives higher value to the two large national parks in the northeastern region.

Runs including connectivity between forest types (“coarseCon”, “coarseClassCon”, “detailClassCon”) display very similar rank priority patterns compared to runs without connectivity. Regions dense with high-priority areas (Fig 4B, 4D and 4F) receive higher overall priority because of the connectivity effect, which is evident from the marginal plots in Fig 4B, 4D and 4F.

### Spatial overlap of priority intervals

**Fig 5:**
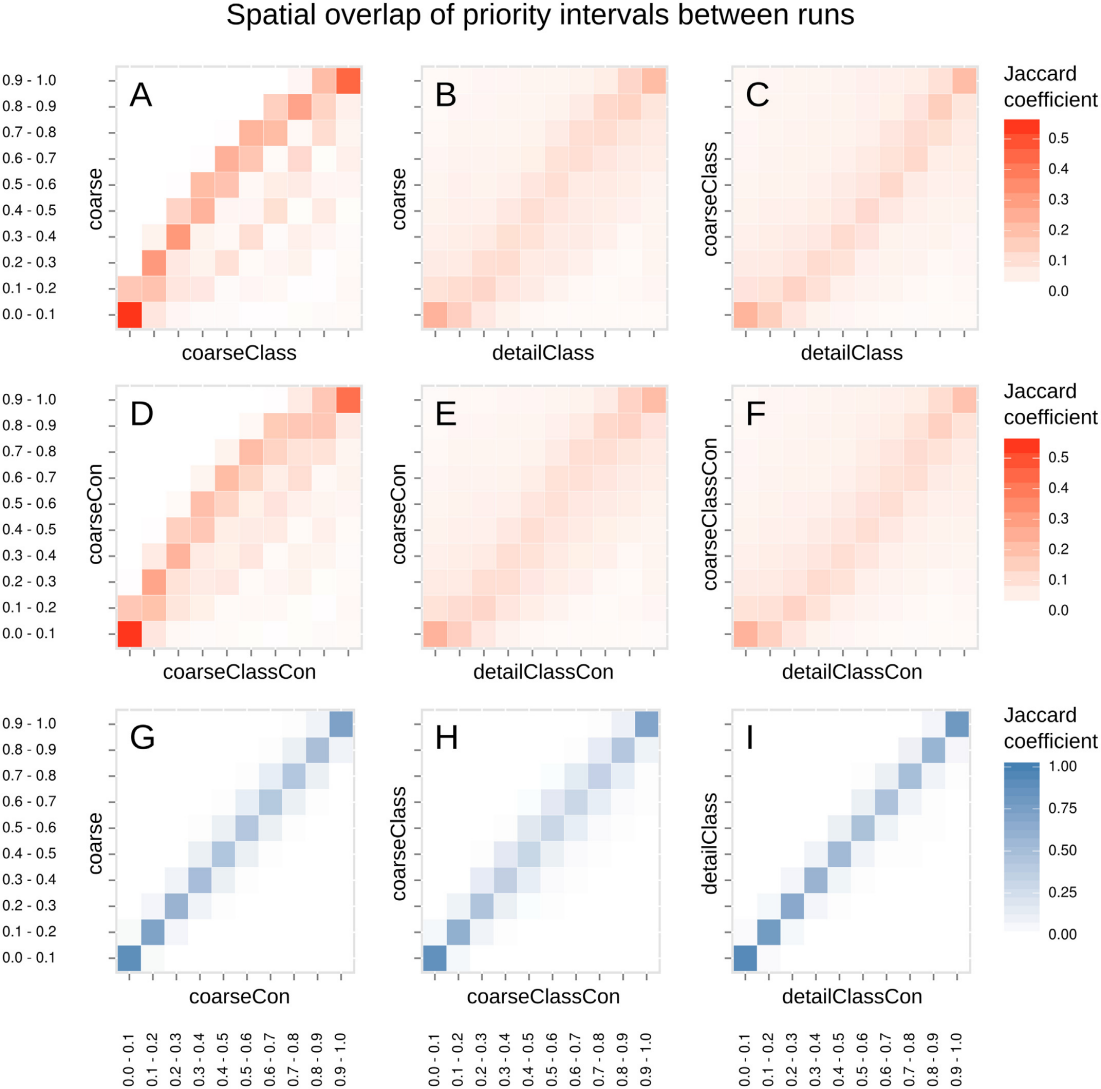
Spatial overlap of 10% priority intervals of between selected pairs of analyses, measured by the Jaccard coefficient. An overlap of 1.0 indicates complete match, whereas overlap of 0.0 means absence of overlap. Panels A-F show comparisons between runs based on different input data sets. Panels G-I show comparisons between analyses that used the same input data, but with and without connectivity. Note that the scale is different for panels A-F and G-I.

Comparing priority intervals between solutions shows the effect of using the site fertility classification. Fig 5A displays an asymmetrical pattern of overlap between priority intervals in “coarse” and “coarseClass”. Large areas in “coarseClass” receive slightly lower priorities than in “coarse”, which is balanced by a small set of areas having significantly higher ranks in “coarseClass” than in “coarse”. There is little overlap between high priorities in “coarse” and low priorities in “coarseClass” (upper-left part of panel 5A) whereas the inverse is different: there is some overlap with relatively high priorities in “coarseClass” and low priorities in “coarse” (lower-right part of panel 5A). The classification of the data causes these overlapping patterns in priority intervals. More specifically, some site fertility classes (most notably the herb-rich and xeric types) are rarer than others and consequently receive more emphasis in the Zonation analysis. This is because Zonation will give priority to features that are rare to begin with.

The best and worst 10% of the priorities have the largest spatial overlaps in all comparisons. Since data classification is the only difference between “coarse” and “coarseClass”, their overall similarity is larger which also explains the higher overlap of the best and worst 10% of priorities (Fig 5A). The overlap is smaller for the best and worst 10% of priorities between “coarse”/“detailClass” and “coarse”/“coarseClass”, but still those overlaps are higher than for the rest of the priority intervals. In other words, the best and the worst areas are more similar between all the analyses even if the underlying input data sets are different.

Fig 5D–5F show the spatial overlaps between runs that account for connectivity. Patterns are similar to the patterns in the runs not accounting for connectivity (Fig 5A–5C) runs with the difference that the patterns are smoother and more aggregated in all comparisons (Fig 5D–5F). Comparisons between runs based on the same input data set with and without accounting for connectivity (Fig 5G–5I) show a strong overlap between the same priority intervals in different runs. The overlap tends to increase when moving towards the highest or lowest priority areas of the study area. This reaffirms that connectivity as defined in this study has an effect only on a local scale.

**Fig 6:**
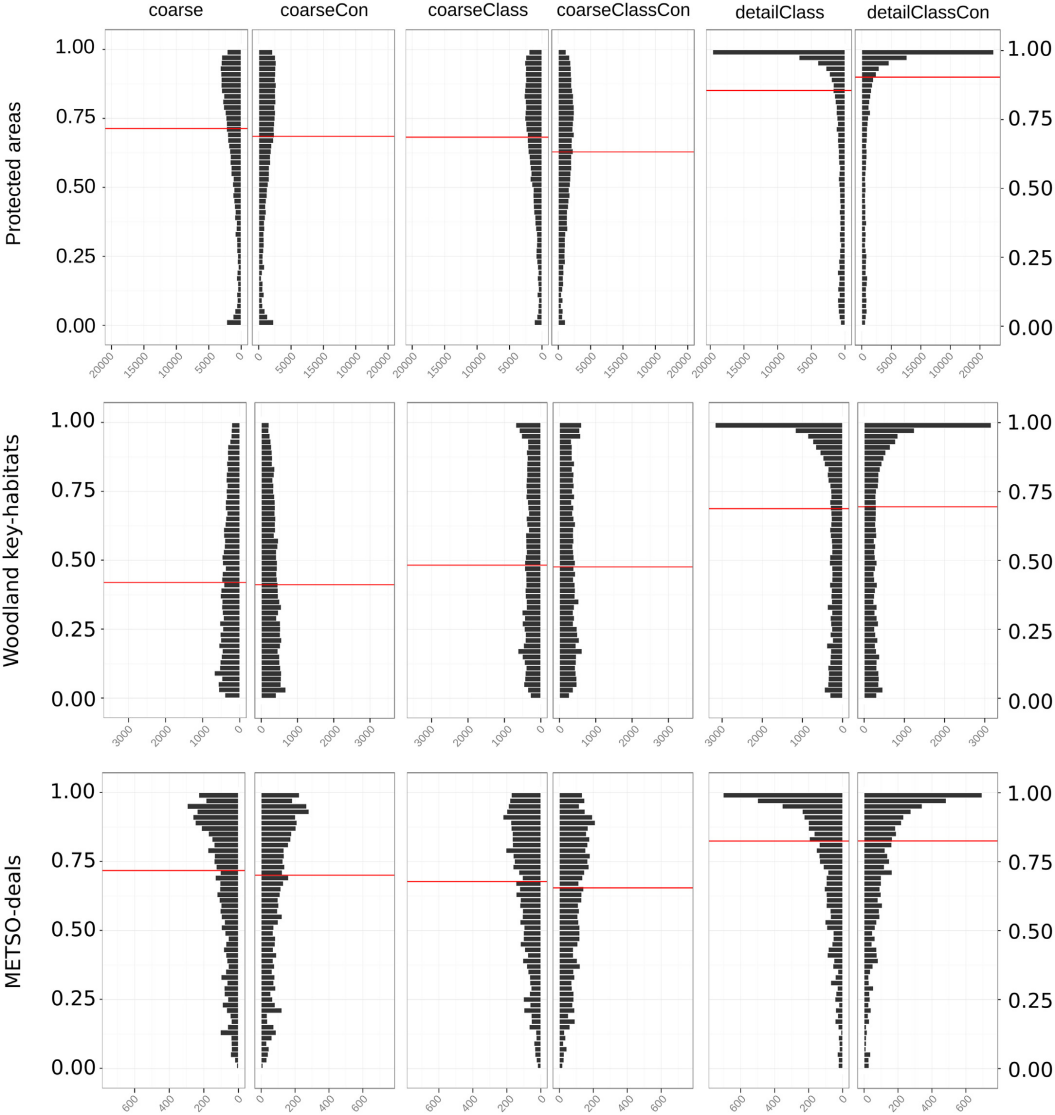
Distribution of priority ranks within areas of known conservation value. The columns in each panel show the difference between variants with (left) and without connectivity (right). All spatial validation data should on average have higher conservation value than the surrounding forests, which mostly have a history of economically motivated management. Red horizontal line corresponds to the median value.

Comparison to spatial validation dataProtected areas have relatively high median priorities in all runs (Fig 6). “detailClass” and “detailClassCon” have the highest median priorities within the protected areas (~0.85 and ~0.90), followed by “coarse” and “coarseCon” (~0.71 and ~0.69). Woodland key-habitats also have quite high median priorities in solutions “detailClass” and “detailClassCon” (both ~0.69), but the distribution of priorities is not as skewed as with protected areas. For “coarseClass” and “coarseClassCon”, WKHs have a median priority of ~0.48, and the median values are even lower for “coarse” and “coarseCon” (~0.42 and ~0.41). Locations admitted to the METSO programme receive the highest median priorities values in “detailClass” and “detailClassCon” (both ~0.82).“coarse” and “coarseCon” have a median priority value similar to those of protected areas (~0.72 and ~0.70), as do “coarseClass” and “coarseClassCon” (~0.68 and ~0.65). In all cases, the difference between runs with and without connectivity is small, except in the case of protected areas. Overall solutions “detailClass” and “detailClassCon” perform better than the others, potentially indicating higher accuracy of the more detailed data and demonstrating the utility of using detailed data from on-the-ground forest inventories.

### Feature representation

**Fig 7:**
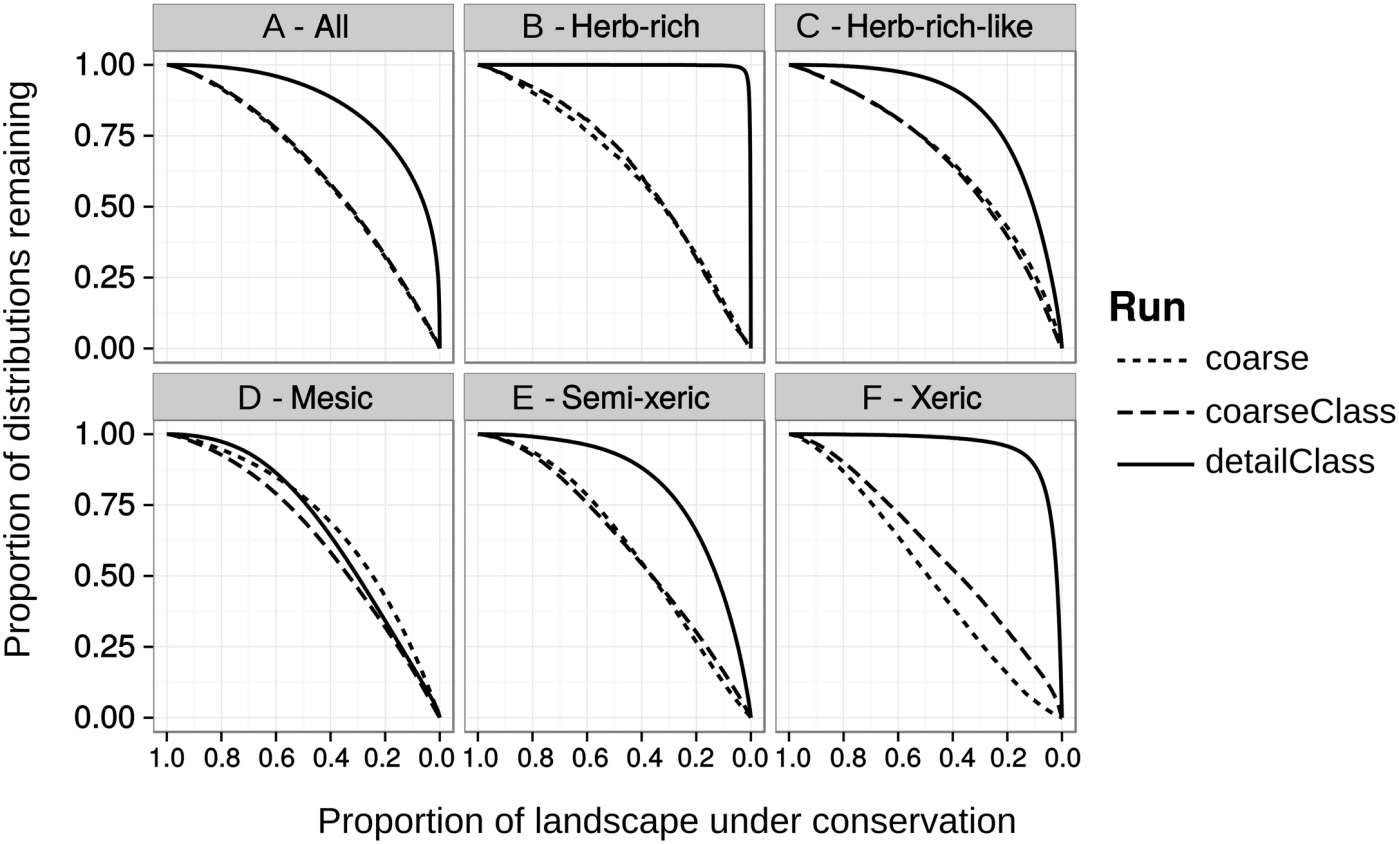
The performance of solutions based on coarser data measured by their ability to cover features in the detailed data. The performance curves show for each site fertility class the mean occurrence levels of biodiversity features in the detailed data. The solid curves are for “detailClass”, which uses detailed data. The dotted (“coarse”) and dashed (“coarseClass”) represent coarse data solutions, and show how much representation of the detailed—and presumably more accurate—data would be lost if the prioritization was based on coarser data. The same comparison between “coarseCon”, “coarseClassCon”, and “detailClassCon” produced very similar results (not shown).

Loading the priority rank order from the runs based on coarse input data (“coarse” and “coarseClass”) revealed differences in performance. Fig 7 shows the overall performance, i.e. how much of the initial representation levels from the detailed data can be covered by protecting a given fraction of the landscape. Fig 7A shows that on average, priority rankings “coarse” and “coarseClass” perform much worse than “detailClass”. For example, protecting the best 10% of the landscape using the ranking from “detailClass” would cover on average approximately 54% of the original distributions of all features from the detailed input data set. In comparison, solutions “coarse” and “coarseClass” would cover on average only ~15% and ~16% of the features in the detailed data, respectively (Fig 7A). This difference is even more pronounced when examining the solutions that use additional site fertility classes. For example, the best 10% of the landscape under “detailClass” covers ~93% of features in herb-rich sites, whereas solutions “coarse” and “coarseClass” only achieve a coverage of ~15% and ~14%, respectively (Fig 7B). For all other site fertility classes except for mesic, the performance of “detailClass” is superior to that of “coarse” and “coarseClass”. The performance levels of runs that account for connectivity (“coarseCon”, “coarseClassCon”, “detailClassCon”) are omitted here, because they are very similar to those of “coarse”, “coarseClass”, and “detailClass”.

## Discussion

### Can forest inventory data be used to identify valuable areas for conservation?

Our results demonstrate that 1) inventory data collected primarily for operational forest planning is informative for spatial conservation prioritization, and 2) openly available remote-sensing based data performs reasonably well for large mature forest areas, but fails to detect valuable sites of smaller size. Therefore, if the spatial prioritization includes objectives for detecting small scale biodiversity feature occurrences such as the WKHs, a more detailed input data are needed.

On the scale of the whole study area, priority patterns between runs based on the coarse and detailed data are relatively similar, but have at least three key differences. First, analyses based on the coarser data give higher priority to a large area at the southwestern part of the province. This is because the MS-NFI data has high estimated values for birch and other deciduous trees in the region, which also has a high incidence of fertile soils. Deciduous trees and fertile site types are less common than other tree species and site fertility types. They furthermore have higher weights assigned in the Zonation analysis due to relatively high associated biodiversity values (see also S1 Appendix). Second, analyses based on the more detailed data give existing large protected areas even much higher priorities. This is most probably because, compared to the coarse data, the detailed data available from within protected areas describes more accurately the mature stands within the PAs. Third, since the detailed data have information also on the occurrence of small but valuable forest (e.g. herb-rich sites or mature deciduous trees) that is not correctly represented in the coarse data, the high-priority sites are more evenly distributed over the whole study area (see the marginal plots in Fig 4).

Of the three validation data sets, WKHs have the smallest average size per site and the most fine-grained structural features important for biodiversity. The coarse data is simply unable to pick up such features. This is not surprising as the coarse data we are using (MS-NFI) is known to have low statistical precision for small area estimates [63,97]. Of course, when available, information about WKHs can be included in the prioritization process itself. We did not do so here, because that would have excluded the use of WKH data as an external validation source.

Extent and resolution are important factors in analyses that account for connectivity. The small effect connectivity has on the priority rank distributions within the validation data sets may appear surprising, especially since the effect of connectivity is quite pronounced over larger areas (Fig 4). However, even when combined the validation data sets cover only a small fraction of the total landscape (2.5%, Table 1) and the mean decay distance for dispersal we used (2 km) is relatively large compared to the average size of sites in the validation data. For these reasons, accounting for connectivity actually decreases the median priority for all other validation data sets except the protected areas, which are larger and thus by definition better connected internally.

The validation procedure we have used relies on a few key assumptions. First, we assume that the indices we have constructed truly reflect conservation value. While we have not validated the indices against actual species occurrence data, features we have emphasized in the construction of the index are important for biodiversity in the Finnish boreal forest (see e.g. [74,75]). Second, we assume that the validation data sets actually describe locations of high conservation value, and that they should therefore receive higher than average priority in spatial prioritization analyses. Protected areas have traditionally been established on less productive soils [59,98] and they usually do not represent the full spectrum of species or habitats in any given region. However, being set aside from the prevailing forest management regimes will over time lead to a less even forest structure [99], thereby accumulating important resources such as dead-wood [100]. METSO-sites are on average smaller than many of the existing protected areas, but because of the stringent selection criteria and on-ground evaluation of each site, their ecological quality is high and studies have shown that they do indeed have higher species richness and that they contain more rare and threatened species than their surrounding areas [84]. WKHs are scattered more evenly over the landscape and according to a recent meta-analysis [80] they contain elevated amounts of critical resources (dead-wood, etc.) that support a comparatively large number of species. However, the average size of a WKH site is small (0.67 ha in Finland [101]), meaning that their capability to support populations long-term is questionable.

### Trade-offs between different data and prioritization objectives

Conservation scientists, managers, and practitioners are often faced with tight schedules and limited budgets, and thus have to decide whether it is worth the time and money to try to collect more data [102,103]. Collecting more data often includes spending time and money on trying to gain access to more detailed data that may not be openly available. Conservation prioritization based on incomplete data runs the risk of commission and omission errors, selecting sites that are not valuable in reality or missing sites that are [36]. According to our results, the analyses based on coarse and detailed data produce spatial priority patterns that are broadly speaking similar but have differences in more local scale (Fig 5). Top and low priorities are slightly more overlapping than the middle-range. Importantly, however, the high-priorities of any of the analyses do not much overlap with the low priorities in any other run. If they did, using coarse data as basis for prioritization would produce wildly different and often incorrect results.

While coarse data is able to describe broad priority patterns correctly, we found that the less abundant biodiversity features such as herb-rich and xeric forest types are not identified well (Fig 7). For example, if we are interested in the top 10% of the landscape, the prioritization based on coarse data with classes captures only half of the representation of biodiversity features that can be achieved if using detailed data. Even if the high-priority areas have a large overlap spatially, using the coarser data misses much of the occurrences of herb-rich sites and woodland key habitats.

The differences between the analyses based on the coarse and coarse with classes input data sets are particularly interesting, as it is temptingly practical to improve existing data with simple classification scheme. The inclusion of the classification does slightly improve the performance for rarer classes (Fig 7) so everything else being equal, an ecologically-justified classification of the data can improve the results.

Including connectivity in the analysis raises the priority of regions that have high quality sites at high densities, thus identifying regions where metapopulations might be able to persist. This is particularly important for many threatened forest species that suffer from habitat loss and fragmentation [104–106]. However, emphasizing connectivity will happen at the expense of individual high-quality sites that are relatively isolated [13,26]. Increasing the priority of medium-quality and well-connected forests will lower the priority of other locally similar sites and possibly even poorly connected high-quality sites (Fig 4). Including connectivity will also emphasize large, overall high-quality areas such as protected areas (Fig 6).

### Opening up forest inventory data is an opportunity for integrated forest and conservation planning in the boreal zone

Open forest inventory data has a major role in conservation planning and decision-making in the boreal region. It enables equal and inclusive access to the best available data [107], it makes the supporting scientific analysis more transparent, and it enhances the repeatability of the whole conservation planning process [40]. Repeatability is especially important for applied research supporting decision-making, because underlying objectives may change, old data are updated, and new information can accumulate rapidly. Transparency and repeatability are also important for the process of translating regional plans into local conservation action: whereas regional plans incorporate important factors such connectivity and the representativeness of the protected area network as a whole, local action can be understood as individual management actions that sometimes unfortunately are poorly linked to regional planning [93]. Plugging into regional and local forest planning through the use of forest inventory data presents new opportunities for conservation prioritization especially in countries of the boreal zone which already have sophisticated forest planning and inventory systems in place.

In summary, we have shown that coarse, NFI-derived data works reasonably well in the identification of broad spatial conservation priorities, but we also found that more detailed inventory data is needed to capture the structural attributes at the local-scale. While it is encouraging to see that inventory data is becoming more openly available, conservation research and decision-making would benefit from more open data policies especially in government organizations. The approach we have taken in this work builds upon previously published work [13,74,75] and methodology [20]. Here we make all analysis implementations (see S1 Appendix) and data (where possible, see 2.3) available to enable others to adapt the approach for their own uses. The approach described here is being used in the implementation of the Finnish national forest conservation programme, and we continue our efforts to improve the approach.

## Supporting Information

S1 Appendix Supporting information on the data and data pre-processing. (DOCX)

S1 Text Granted, explicit and written permission to publish Figs 1, 2 and 3 under a CC BY license by the Finnish Forest Centre. (DOCX)

S1 Fig The expert-derived benefit functions for conservation value. Benefit functions are used to scale the perceived, expert-derived conservation value (y-axis) to structural characteristics of the forest (x-axis). These functions are specific to tree species groups.
(TIF)

S1 Table Biodiversity feature weights used in the Zonation analyses. Features 1–4 are specific to runs “coarse” and “coarseCon”, while features 5–24 are the forest types defined in Material & Methods.
(DOCX)

S2 Table Matrix connectivity multipliers used in Zonation runs “coarse” and “coarseCon”. The numbers on the header row and column correspond to the feature IDs in S1 Table. Note that the matrix is asymmetrical, i.e. the direction of the connectivity effect matters. Columns represent the forest types causing the connectivity effect, rows the forest types receiving the connectivity effect.
(DOCX)

S3 Table Matrix connectivity multipliers used in Zonation runs “coarseClass”, “coarseClassCon”, “detailClass” and “detailClassCon”. The numbers on the header row and column correspond to the feature IDs in S1 Table. Note that the matrix is asymmetrical, i.e. the direction of the connectivity effect matters. Columns represent the forest types afflicting the connectivity effect, rows the forest types receiving the connectivity effect.
(DOCX)

## References

[1] Game ET, Meijaard E, Sheil D, MacDonald-Madden E. Conservation in a wicked complex world; challenges and solutions. Conserv Lett. 2014;7: 271–277. 10.1111/conl.12050

[2] Reyers B, Roux DJ, Cowling RM, Ginsburg AE, Nel JL, O’Farrell P. Conservation planning as a transdisciplinary process. Conserv Biol. 2010;24: 957–65. 10.1111/j.1523-1739.2010.01497.x 20345401

[3] Pooley SP, Mendelsohn JA, Milner-Gulland EJ. Hunting down the chimera of multiple disciplinarity in conservation science. Conserv Biol. 2013;28: 22–32. 10.1111/cobi.12183 24299167 PMC4232892

[4] Keane A. Unusual data in conservation science: searching for validation. Anim Conserv. 2013;10: 604–605. 10.1111/acv.12091

[5] Ferrier S, Drielsma M. Synthesis of pattern and process in biodiversity conservation assessment: a flexible whole-landscape modelling framework. Divers Distrib. 2010;16: 386–402. 10.1111/j.1472-4642.2010.00657.x

[6] Ferrier S, Wintle BA. Quantitative approaches to spatial conservation prioritization: matching the solution to the need In: Moilanen A, Wilson KA, Possingham HP, editors. Spatial conservation prioritization: quantitative methods & computational tools. Oxford: Oxford University Press; 2009 p. 304.

[7] Naidoo R, Balmford A, Ferraro PJ, Polasky S, Ricketts TH, Rouget M. Integrating economic costs into conservation planning. Trends Ecol Evol. 2006;21: 681–687. 10.1016/j.tree.2006.10.003 17050033

[8] Branquart E, Verheyen K, Latham J. Selection criteria of protected forest areas in Europe: the theory and the real world. Biol Conserv. 2008;141: 2795–2806. 10.1016/j.biocon.2008.08.015

[9] Scheller RM, Mladenoff DJ. An ecological classification of forest landscape simulation models: tools and strategies for understanding broad-scale forested ecosystems. Landsc Ecol. 2007;22: 491–505. 10.1007/s10980-006-9048-4

[10] Kujala H, Moilanen A, Araújo MB, Cabeza M. Conservation planning with uncertain climate change projections. PLoS One. 2013;8: 1–12. 10.1371/journal.pone.0053315 PMC3566137 23405068

[11] Visconti P, Pressey RL, Segan DB, Wintle BA. Conservation planning with dynamic threats: the role of spatial design and priority setting for species’ persistence. Biol Conserv. 2010;143: 756–767.

[12] Pouzols FM, Toivonen T, Di Minin E, Kukkala AS, Kullberg P, Kuusterä J, et al Global protected area expansion is compromised by projected land-use and parochialism. Nature. 2014;516: 383–386. 10.1038/nature14032 25494203

[13] Arponen A, Lehtomäki J, Leppänen J, Tomppo E, Moilanen A. Effects of connectivity and spatial resolution of analyses on conservation prioritization across large extents. Conserv Biol. 2012;26: 294–304. 10.1111/j.1523-1739.2011.01814.x 22268786

[14] Moilanen A, Wilson KA, Possingham HP. Spatial conservation prioritization: quantitative methods and computational tools. Oxford, UK: Oxford University Press; 2009.

[15] Kukkala AS, Moilanen A. Core concepts of spatial prioritisation in systematic conservation planning. Biol Rev. 2012;88: 443–464. 10.1111/brv.12008 23279291 PMC3654170

[16] Pressey RL, Cabeza M, Watts ME, Cowling RM, Wilson KA. Conservation planning in a changing world. Trends Ecol Evol. 2007;22: 583–592. 10.1016/j.tree.2007.10.001 17981360

[17] Wilson KA, Underwood EC, Morrison SA, Klausmeyer KR, Murdoch WW, Reyers B, et al Conserving biodiversity efficiently: what to do, where, and when. Mace GM, editor. PLoS Biol. 2007;5: 12 10.1371/journal.pbio.0050223 PMC1950771 17713985

[18] Knight AT, Rodrigues ASL, Strange N, Tew T, Wilson KA. Designing effective solutions to conservation planning problems In: Macdonald DW, Willis KJ, editors. Key topics in conservation boilogy 2. Oxford: Blackwell-Wiley; 2013 pp. 362–383.

[19] Moilanen A, Possingham HP, Polasky S. A mathematical classification of conseravation prioritization problems In: Moilanen A, Wilson K, Possingham HP, editors. Spatial conservation prioritization: quantitative methods & computational tools. Oxford: Oxford University Press; 2009 pp. 28–42.

[20] Lehtomäki J, Moilanen A. Methods and workflow for spatial conservation prioritization using Zonation. Environ Model Softw. 2013;47: 128–137. 10.1016/j.envsoft.2013.05.001

[21] Moilanen A, Kujala H, Leathwick JR. The Zonation framework and software for conservation prioritization In: Moilanen A, Wilson KH, Possingham HP, editors. Spatial conservation prioritization: quantitative methods & computational toolsnservation Prioritization. Oxford University Press; 2009 pp. 196–210.

[22] Pressey RL, Watts ME, Barrett TW, Ridges MJ. The C-Plan conservation planning system: origins, applications and possible futures In: Moilanen A, Wilson K, Possingham HP, editors. Spatial conservation prioritization: quantitative methods & computational tools. Oxford University Press; 2009 pp. 211–234.

[23] Ciarleglio M, Barnes JW, Sarkar S. ConsNet: new software for the selection of conservation area networks with spatial and multi-criteria analyses. Ecography (Cop). 2009;32: 205–209. 10.1111/j.1600-0587.2008.05721.x

[24] Possingham HP, Ball IR, Andelman SJ. Mathematical methods for identifying representative reserve networks In: Ferson S, Burgman MA, editors. Quantitative methods for conservation biology. New York: Springer-Verlag; 2000 pp. 291–305.

[25] Wilson KA, Cabeza M, Klein CJ. Fundamental concepts of spatial conservation prioritization In: Moilanen AJ, Wilson KA, Possingham HP, editors. Spatial conservation prioritization: quantitative methods & computational tools. Oxford: Oxford University Press; 2009 pp. 16–27.

[26] Hodgson JA, Thomas CD, Wintle BA, Moilanen A. Climate change, connectivity and conservation decision making: back to basics. J Appl Ecol. 2009;46: 964–969. 10.1111/j.1365-2664.2009.01695.x

[27] Hanski I. Metapopulation dynamics. Nature. 1998;396: 41–49.

[28] Rayfield B, Fortin M-J, Fall A. Connectivity for conservation: a framework to classify network measures. Ecology. 2011;92: 847–858. 10.1890/09-2190.1 21661548

[29] Williams JC, Revelle CS, Levin SA. Spatial attributes and reserve design models: a review. Environ Model Assess. 2005;10: 163–181. 10.1007/s10666-005-9007-5

[30] Heller NE, Zavaleta ES. Biodiversity management in the face of climate change: Aareview of 22 years of recommendations. Biol Conserv. 2009;142: 14–32.

[31] Kool JT, Moilanen A, Treml EA. Population connectivity: recent advances and new perspectives. Landsc Ecol. 2013;28: 165–185. 10.1007/s10980-012-9819-z

[32] Langford WT, Gordon A, Bastin L, Bekessy SA, White MD, Newell G. Raising the bar for systematic conservation planning. Trends Ecol Evol. 2011;26: 634–640. 10.1016/j.tree.2011.08.001 21899914

[33] Pettorelli N, Laurance WF, O’Brien TG, Wegmann M, Nagendra H, Turner W. Satellite remote sensing for applied ecologists: opportunities and challenges. J Appl Ecol. 2014;51: 839–848. 10.1111/1365-2664.12261

[34] Maeda EE, Torres JA. Open environmental data in developing countries: who benefits? Ambio. 2012;41: 410–2. 10.1007/s13280-012-0283-4 22528983 PMC3393066

[35] Pressey RL. Conservation planning and biodiversity: assembling the best data for the job. Conserv Biol. 2004;18: 1677–1681. 10.1111/j.1523-1739.2004.00434.x

[36] Rondinini C, Wilson K a, Boitani L, Grantham H, Possingham HP. Tradeoffs of different types of species occurrence data for use in systematic conservation planning. Ecol Lett. 2006;9: 1136–1145. 10.1111/j.1461-0248.2006.00970.x 16972877

[37] Boitani L, Maiorano L, Baisero D, Falcucci A, Visconti P, Rondinini C. What spatial data do we need to develop global mammal conservation strategies? Philos Trans R Soc B Biol Sci. 2011;366: 2623–2632. 10.1098/rstb.2011.0117 PMC3140738 21844041

[38] Grantham HS, Pressey RL, Wells JA, Beattie AJ. Effectiveness of biodiversity surrogates for conservation planning: different measures of effectiveness generate a kaleidoscope of variation. Moen J, editor. PLoS One. 2010;5: 1–12. 10.1371/journal.pone.0011430 PMC2904370 20644726

[39] Carpenter SR, Armbrust EV, Arzberger PW, Iii FSC, Elser JJ, Hackett EJ, et al Accelerate synthesis in ecology and environmental sciences. Bioscience. 2009;59: 699–701. 10.1525/bio.2009.59.8.11

[40] Uhlir PF, Schröder P. Open data for global science: a review of recent developments in national and international scientific data policies and related proposals. Data Sci J. 2007;6: 36–53. 10.2481/dsj.6.OD1

[41] Wolkovich EM, Regetz J, O’Connor MI. Advances in global change research require open science by individual researchers. Glob Chang Biol. 2012;18: 2102–2110. 10.1111/j.1365-2486.2012.02693.x

[42] Egloff W, Patterson DJ, Agosti D, Hagedorn G. Open exchange of scientific knowledge and European copyright: the case of biodiversity information. Zookeys. 2014;135: 109–135. 10.3897/zookeys.414.7717 PMC4086052 25009418

[43] Arzberger P, Schroeder P, Beaulieu A, Bowker G, Casey K, Laaksonen L, et al An international framework to promote access to data. Science. 2004;303: 1777–1778. 10.1126/science.1095958 15031482

[44] Bode M, Wilson KA, Brooks TM, Turner WR, Mittermeier RA, Mcbride MF, et al Cost-effective global conservation spending is robust to taxonomic group. Proc Natl Acad Sci U S A. 2008;105: 6498–501. 10.1073/pnas.0710705105 18413614 PMC2359771

[45] Reichman OJ, Jones MB, Schildhauer MP. Challenges and opportunities of open data in ecology. Science. 2011;331: 703–705. 10.1126/science.1197962 21311007

[46] Pullin AS, Salafsky N. Save the whales? Save the rainforest? Save the data! Conserv Biol. 2010;24: 915–7. 10.1111/j.1523-1739.2010.01537.x 20636614

[47] Bradshaw CJ, Warkentin IG, Sodhi NS. Urgent preservation of boreal carbon stocks and biodiversity. Trends Ecol Evol. 2009;24: 541–8. 10.1016/j.tree.2009.03.019 19679372

[48] Kuuluvainen T, Grenfell R. Natural disturbance emulation in boreal forest ecosystem management—theories, strategies, and a comparison with conventional even-aged management. Can J For Res. 2012;1203: 1185–1203.

[49] Mönkkönen M. Managing Nordic boreal forest landscapes for biodiversity: ecological and economic perspectives. Biodivers Conserv. 1999;8: 85–99. 10.1023/A:1008813225086

[50] Stephens SL, Burrows N, Buyantuyev A, Gray RW, Keane RE, Kubian R, et al Temperate and boreal forest mega-fires: characteristics and challenges. Front Ecol Environ. 2014;12: 115–122. 10.1890/120332

[51] Halme P, Allen KA, Auninš A, Bradshaw RHW, Brumelis G, Cada V, et al Challenges of ecological restoration: lessons from forests in northern Europe. Biol Conserv. 2013;167: 248–256. 10.1016/j.biocon.2013.08.029

[52] Esseen P, Ehnström B, Ericson L, Sjöberg K. Boreal forests. Ecol Bull. 1997;46: 16–47.

[53] Mace GM, Masundire H, Baillie JEM. Chapter 4: Biodiversity In: Hassan R, Scholes RJ, Ash N, editors. Ecosystems and human well-being: current state and trends, Volume 1 Washington D.C.: Island Press; 2005 pp. 79–122.

[54] Puumalainen J, Kennedy P, Folving S. Monitoring forest biodiversity: a European perspective with reference to temperate and boreal forest zone. J Environ Manage. 2003;67: 5–14. 10.1016/S0301-4797(02)00183-4 12659799

[55] Esseen P, Ehnström B, Ericson L, Sjöberg K. Boreal forests—the focal habitats of Fennoscandia In: Hansson L, editor. Ecological principles of nature conservation. London: Elsevier; 1992 pp. 252–325.

[56] Hanski I. Extinction debt and species credit in boreal forests: modelling the consequences of different approaches to biodiversity conservation. Ann Zool Fennici. 2000;37: 271–280.

[57] Martikainen P, Siitonen J, Punttila P, Kaila L, Rauh J. Species richness of Coleoptera in mature managed and old-growth boreal forests in southern Finland. Biol Conserv. 2000;94: 199–209. 10.1016/S0006-3207(99)00175-5

[58] Moen J, Rist L, Bishop K, Chapin FS III, Ellison D, Petersson H, et al Eye on the Taiga: removing global policy impediments to safeguard the boreal forest. Conserv Lett. 2014;7: 408–418. 10.1111/conl.12098

[59] Elbakidze M, Angelstam PK, Sobolev N, Degerman E, Andersson K, Axelsson R, et al Protected area as an indicator of ecological sustainability? A century of development in Europe’s boreal forest. Ambio. 2013;42: 201–214. 10.1007/s13280-012-0375-1 23475656 PMC3593037

[60] Andrew ME, Wulder MA, Coops NC. Identification of de facto protected areas in boreal Canada. Biol Conserv. 2012;146: 97–107. 10.1016/j.biocon.2011.11.029

[61] Chirici G, Mcroberts RE, Winter S, Bertini R, Bra U, Asensio IA, et al National forest inventory contributions to forest biodiversity monitoring. For Sci. 2012;58: 257–268. 10.5849/forsci.12-003

[62] Tomppo EO, Haakana M, Kaitila M, Peräsaari J. Multi-source national forest inventory—methods and applications. Dordrecht: Springer; 2008.

[63] Corona P, Chirici G, Mcroberts RE, Winter S, Barbati A. Contribution of large-scale forest inventories to biodiversity assessment and monitoring. For Ecol Manage. 2011;262: 2061–2069. 10.1016/j.foreco.2011.08.044

[64] Winter S, Chirici G, McRoberts E, Hauk E, Tomppo E. Possibilities for harmonizing national forest inventory data for use in forest biodiversity assessments. Forestry. 2008;81 10.1093/forestry/cpm042

[65] Kallio AM, Hänninen R, Vainikainen N, Luque S. Biodiversity value and the optimal location of forest conservation sites in Southern Finland. Ecol Econ. 2008;67: 232–243. 10.1016/j.ecolecon.2008.05.005

[66] Chirici G, Winter S, McRoberts RE. National forest inventories: contributions to forest biodiversity assessments [Internet]. Dordrecht: Springer; 2011 Available: http://link.springer.com/book/10.1007/978-94-007-0482-4

[67] Tomppo E, Gschwantner T, Lawrence M, McRoberts RE. National forest inventories—pathways for common reporting Media. Dordrecht: Springer; 2010.

[68] McElhinny C, Gibbons P, Brack C, Bauhus J. Forest and woodland stand structural complexity: its definition and measurement. For Ecol Manage. 2005;218: 1–24. 10.1016/j.foreco.2005.08.034

[69] Finnish Government. Government Resolution on the Forest Biodiversity Programme for Southern Finland 2008–2016 (METSO) [Internet]. 2008 [cited 3 May 2014] p. 15. Available: http://www.mmm.fi/attachments/metsat/5yckfcmWR/METSOResolution2008-2016_ENGL.pdf

[70] Guisan A, Tingley R, Baumgartner JB, Naujokaitis-Lewis I, Sutcliffe PR, Tulloch AIT, et al Predicting species distributions for conservation decisions. Ecol Lett. 2013;16: 1424–1435. 10.1111/ele.12189 24134332 PMC4280402

[71] Finnish Forest Research Institute. Finnish statistical yearbook of forestry. Vantaa: Metla; 2013.

[72] Tuominen S, Balazs A, Korhonen KT, Muinonen E. NFI plots as complementary reference data in forest inventory based on airborne laser scanning and aerial photography in Finland. Silva Fenn. 2014;48: 1–20. 10.14214/sf.983

[73] Tomppo EO. The Finnish national forest inventory In: Kangas A, Maltamo M, editors. Forest inventory: methodology and applications. Managing F. Dordrecht: Springer; 2006 pp. 179–194.

[74] Lehtomäki J, Tomppo E, Kuokkanen P, Hanski I, Moilanen A. Applying spatial conservation prioritization software and high-resolution GIS data to a national-scale study in forest conservation. For Ecol Manage. 2009;258: 2439–2449. 10.1016/j.foreco.2009.08.026

[75] Sirkiä S, Lehtomäki J, Lindén H, Tomppo E, Moilanen A. Defining spatial priorities for capercaillie Tetrao urogallus lekking landscape conservation in south-central Finland. Wildlife Biol. 2012;18: 337–353. 10.2981/11-073

[76] Finnish Forest Research Institute. Multi-source national forest inventory (MS-NFI) [Internet]. 2014 [cited 4 May 2014]. Available: http://www.metla.fi/ohjelma/vmi/vmi-moni-en.htm

[77] Nagendra PM, Goldberg M. Image segmentation with directed trees. IEEE Trans Pattern Anal Mach Interligence. 1980;1: 185–191. 10.1109/TPAMI.1980.4766999 21868892

[78] Pekkarinen A. Image segment-based spectral features in the estimation of timber volume. Remote Sens Environ. 2002;82: 349–359. 10.1016/S0034-4257(02)00052-4

[79] Mönkkönen M, Juutinen A, Mazziotta A, Miettinen K, Podkopaev D, Reunanen P, et al Spatially dynamic forest management to sustain biodiversity and economic returns. J Environ Manage. 2014;134C: 80–89. 10.1016/j.jenvman.2013.12.021 24463852

[80] Timonen J, Gustafsson L, Kotiaho JS, Mönkkönen M. Hotspots in cold climate: conservation value of woodland key habitats in boreal forests. Biol Conserv. 2011;144: 2061–2067. 10.1016/j.biocon.2011.02.016

[81] Aune K, Jonsson BG, Moen J, Gunnar B. Isolation and edge effects among woodland key habitats in Sweden: is forest policy promoting fragmentation? Biol Conserv. 2005;124: 89–95. 10.1016/j.biocon.2005.01.015

[82] Pykälä J, Heikkinen RK, Toivonen H, Jääskeläinen K. Importance of Forest Act habitats for epiphytic lichens in Finnish managed forests. For Ecol Manage. 2006;223: 84–92. 10.1016/j.foreco.2005.10.059

[83] Korhonen K, Hujala T, Kurttila M. Diffusion of voluntary protection among family forest owners: decision process and success factors. For Policy Econ. 2013;26: 82–90. 10.1016/j.forpol.2012.08.010

[84] Siitonen J, Penttilä R, Ihalainen A. METSO-ohjelman uusien pysyvien ja määräaikaisten suojelualueiden ekologinen laatu Uudenmaan alueella Metsätieteen aikakausikirja. Vantaa; 2012: 259–284. Available: http://www.metla.fi/aikakauskirja/full/ff12/ff124259.pdf

[85] ESRI. ArcGIS Desktop, version 10.2.1 [Internet]. Redlands, CA: Environmental Systems Research Institute; 2014. Available: http://www.esri.com/software/arcgis

[86] Python Development Team. Python Language Reference, version 2.7 [Internet]. Python Software Foundation; 2014. Available: http://www.python.org

[87] GDAL Development Team. GDAL—Geospatial Data Abstraction Library, version 1.10.1 [Internet]. Open Source Geospatial Foundation; 2014. Available: http://www.gdal.org

[88] Moilanen A, Franco AMA, Early RI, Fox R, Wintle BA, Thomas CD. Prioritizing multiple-use landscapes for conservation: methods for large multi-species planning problems. Proc R Soc B Biol Sci. 2005;272: 1885–1891. 10.1098/rspb.2005.3164 PMC1559892 16191593

[89] Moilanen A, Pouzols FM, Meller L, Veach V, Arponen A, Leppänen J, et al. Zonation spatial conservation planning methods and software v. 4, user manual. Helsinki; 2014.

[90] Moilanen A. Reserve selection using nonlinear species distribution models. Am Nat. 2005;165: 695–706. 15937749 10.1086/430011

[91] Arponen A, Kondelin H, Moilanen A. Area-based refinement for selection of reserve sites with the benefit-function approach. Conserv Biol. 2007;21: 527–33. 10.1111/j.1523-1739.2006.00607.x 17391202

[92] Kremen C, Cameron A, Moilanen A, Phillips SJ, Thomas CD, Beentje H, et al Aligning conservation priorities across taxa in Madagascar with high-resolution planning tools. Science. 2008;320: 222–226. 10.1126/science.1155193 18403708

[93] Rayfield B, Moilanen A, Fortin M-J. Incorporating consumer-resource spatial interactions in reserve design. Ecol Modell. 2009;220: 725–733.

[94] Moilanen A. Landscape Zonation, benefit functions and target-based planning: unifying reserve selection strategies. Biol Conserv. 2007;134: 571–579.

[95] R Core Team. R: A Language and Environment for Statistical Computing, version 3.1.0 [Internet]. Vienna, Austria: R Foundation for Statistical Computing; 2014. Available: http://www.r-project.org/

[96] Lehtomäki J. zonator: Utilities for Zonation spatial conservation prioritization software. R package version 0.3.9 [Internet]. Helsinki; 2014. Available: https://github.com/cbig/zonator

[97] Tomppo EO. The Finnish multi-source national forest inventory-small area estimation and map production In: Kangas A, Maltamo M, editors. Forest inventory: methodology and Applications. Dordrecht: Springer; 2006 pp. 195–224.

[98] Scott JM, Davis FW, McGhie RG, Wright RG, Groves C, Estes J. Nature reserves: do they capture the full range of America’s biological diversity? Ecol Appl. 2001;11: 999–1007.

[99] Kuuluvainen T, Tahvonen O, Aakala T. Even-aged and uneven-aged forest management in boreal Fennoscandia: a review. Ambio. 2012;41: 720–37. 10.1007/s13280-012-0289-y 22581386 PMC3472017

[100] Siitonen J, Martikainen P, Punttila P, Rauh J. Coarse woody debris and stand characteristics in mature managed and old-growth boreal mesic forests in southern Finland. For Ecol Manage. 2000;128: 211–225. 10.1016/S0378-1127(99)00148-6

[101] Timonen J, Siitonen J, Gustafsson L, Kotiaho JS, Stokland JN, Sverdrup-Thygeson A, et al Woodland key habitats in northern Europe: concepts, inventory and protection. Scand J For Res. 2010;25: 309–324. 10.1080/02827581.2010.497160

[102] Grantham HS, Wilson KA, Moilanen A, Rebelo T, Possingham HP. Delaying conservation actions for improved knowledge: how long should we wait? Ecol Lett. 2009;12: 293–301. 10.1111/j.1461-0248.2009.01287.x 19243409

[103] Grantham HS, Moilanen A, Wilson KA, Pressey RL, Rebelo TG, Possingham HP. Diminishing return on investment for biodiversity data in conservation planning. Conserv Lett. 2008;1: 190–198. 10.1111/j.1755-263X.2008.00029.x

[104] Nordén J, Penttilä R, Siitonen J, Tomppo E, Ovaskainen O. Specialist species of wood-inhabiting fungi struggle while generalists thrive in fragmented boreal forests. Thrall P, editor. J Ecol. 2013;101: 701–712. 10.1111/1365-2745.12085

[105] Henle K, Davies KF, Kleyer M, Margules C, Settele J. Predictors of species sensitivity to fragmentation. Biodivers Conserv. 2004;13: 207–251. 10.1023/B:BIOC.0000004319.91643.9e

[106] Ranius T, Kindvall O. Extinction risk of wood-living model species in forest landscapes as related to forest history and conservation strategy. Landsc Ecol. 2006;21: 687–698. 10.1007/s10980-005-5222-3

[107] Soranno PA, Cheruvelil KS, Elliott KC, Montgomery GM. It’s good to share: why environmental scientists' ethics are out of date. Bioscience. 2015;65: 69–73. 26955073 10.1093/biosci/biu169 PMC4776715

