# Supplementary material for: What Data to Use for Forest Conservation Planning? A Comparison of Coarse Open and Detailed Proprietary Forest Inventory Data in Finland
Source: PLoS One. 2015 Aug 28;10(8):e0135926. doi: 10.1371/journal.pone.0135926 (PMC4552654; doi:10.1371/journal.pone.0135926)
Supplement: S1 Appendix — (DOCX) [file pone.0135926.s001.docx]

## Data, results, and implementation of data pre-processing and Zonation analysis

The following online repositories contain the data, such results that can be shared, and the implementation of various steps of the study in the form of source code or configuration files. Each repository has its own licensing information.

1. http://dx.doi.org/10.5281/zenodo.14676 - The sources of this manuscript including text, figures and all code needed to analyze the results.
2. http://dx.doi.org/10.5281/zenodo.27240 - Zonation configuration files.

## Data pre-processing

### Segmentation

The segmentation was carried out using a modified implementation of the “segmentation with directed trees” algorithm by Nagendra & Goldberg. The “segmentation with directed trees” aims at detecting regions without using absolute thresholds [1]. The method is based on using an edge image. The algorithm starts by dividing an image to edge and plateau pixels by computing an edge gradient value for each image pixel based on its 3x3 pixel neighborhood. If the edge gradient value was higher than a user-defined gradient threshold value (i.e. indicating high local variation), the pixel was an edge pixel, otherwise a plateau pixel. In the following phases, 1.) the edge pixels linked to the direction of maximum positive edge gradient, and 2.) the plateau pixels are linked to the pixels of the same plateau. A more detailed description of the segmentation algorithm can be found in [2].

Thus, the automatic segmentation process is guided by the local heterogeneity of the input data pixels. In the case when larger segments than produced by initial segmentation are required, separate region merging algorithm can be applied. The region-merging algorithm is based on t-ratio threshold [3] and it is guided by parameters such as the desired minimum size of the final segments and the similarity or dissimilarity of the segments (measured by t-ratio and a user-defined threshold value). Here the size of the output segments was in the range of desired segment size, approx. 1-2 ha, and separate region merging phased was not required.

The stand level variables variables were calculated as average values of the individual pixels within each segment. The variables per tree species were calculated by weighting the pixel level variables by the volumes of individual tree species.

### Habitat quality index

The first component of the model is identifying which are the relevant factors for the prioritization problem at hand followed by the collation of information on the identified factors [4]. We identified the important structural features from the forest inventory databases in several workshops held with experts from Metsähallitus and the FFC. Based on the outcomes of the workshops, we constructed a habitat quality index of the form:

$I_{i}=f\left( diameter \right)\times volume$ (1)

where I is the index value for a cell in location I, and f is a specific sigmoidal function that translates the average diameter (a proxy for maturity) into a multiplier for volume. The rationale behind the sigmoidal shape of the function is that it gives little value to relatively low diameter trees, after which and when approaching a preset inflection point the value increases relatively quickly. Finally, the increase levels off as high enough average diameter values are reached. Again working with experts, we constructed specific sigmoidal functions for each main tree species group (birch, pine, spruce, and other deciduous; see S1 Fig.) which we parameterized differently to reflect how habitat quality increases within each group. For example, other deciduous trees generally are more valuable to conservation at younger age than pine. The functions reflected what the experts generally regarded as valuable for conservation as well as the empirical distribution of average diameter and volume records in the input data.

It is worth noting, that both index formulation presented above are very simple in structure designed to give more value mature forests. Because of the formulation, several forest habitat types, such as rocky outcrops with forests, and several different peatland habitat types, such as spruce mires, might receive relatively low value. Furthermore, relying on volume may give too high value on commercially managed stands.

To construct data sets “Coarse with classes” and “Detailed with classes”, we further split the index rasters into categories based on 5 site fertility classes (herb-rich, herb-rich like, mesic, semi-xeric, and xeric). Therefore, in the end we were left with 4 x 5 = 20 different index rasters.

## Supplementary references

[1] Narendra, P. M., and M. Goldberg. 1980. Image Segmentation with Directed Trees. IEEE Transactions on Pattern Analysis and Machine Interligence **1**:185–191.

[2] Pekkarinen, A. 2002. Image segment-based spectral features in the estimation of timber volume. Remote Sensing of Environment 82:349–359.

[3] Hagner, O. 1990. Computer aided forest stand delineation and inventory based on satellite remote sensing. In: Proceedings of SNS/IUFRO workshop in Umeå 26–28.12.1990: The usability of remote sensing for forest inventory and planning. Swedish University of Agriculture Sciences. Remote Sensing Laboratory. Umeå.

[4] Ferrier S, Wintle BA (2009) Quantitative Approaches to Spatial Conservation Prioritization: Matching the Solution to the Need. In: Moilanen A, Wilson KA, Possingham HP, editors. Spatial Conservation Prioritization: Quantitative Methods & Computational Tools. Oxford: Oxford University Press. p. 304.
