# Supplementary material for: What Data to Use for Forest Conservation Planning? A Comparison of Coarse Open and Detailed Proprietary Forest Inventory Data in Finland
Source: PLoS One. 2015 Aug 28;10(8):e0135926. doi: 10.1371/journal.pone.0135926 (PMC4552654; doi:10.1371/journal.pone.0135926)
Supplement: S1 Text — (DOCX) [file pone.0135926.s002.docx]

Permission is hereby granted by Suomen Metsäkeskus (Finnish Forest Centre) for the open-access journal PLOS ONE to publish Figures 1, 2 and 3 for article "What Data to Use for Forest Conservation planning? A Comparison of Coarse Open and Detailed Proprietary Forest Inventory Data in Finland" under the Creative Commons Attribution License (CCAL) CC BY 3.0.

Contact person:

Antti Leinonen
Finnish Forest Centre (Suomen Metsäkeskus)
Kauppakatu 25 A
87100 Kajaani
Finland
+358 40 841 5790
antti.leinonen@metsakeskus.fi
