# Supplementary figures and images for: What Data to Use for Forest Conservation Planning? A Comparison of Coarse Open and Detailed Proprietary Forest Inventory Data in Finland
Source: PLoS One. 2015 Aug 28;10(8):e0135926. doi: 10.1371/journal.pone.0135926 (PMC4552654; doi:10.1371/journal.pone.0135926)

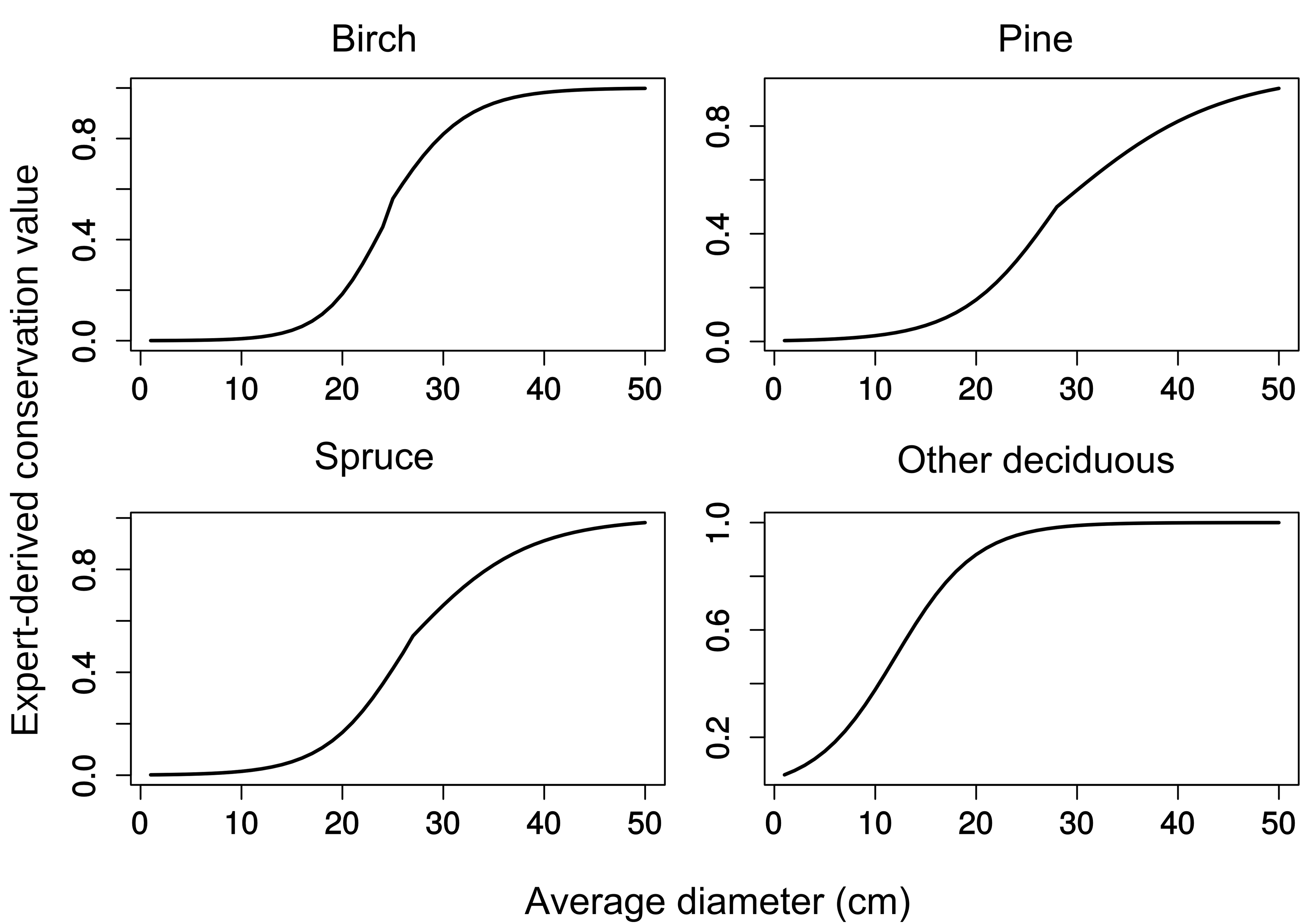

Supplement: S1 Fig — Benefit functions are used to scale the perceived, expert-derived conservation value (y-axis) to structural characteristics of the forest (x-axis). These functions are specific to tree species groups. (TIF) [file pone.0135926.s003.tif]
