# Supplementary material for: What Data to Use for Forest Conservation Planning? A Comparison of Coarse Open and Detailed Proprietary Forest Inventory Data in Finland
Source: PLoS One. 2015 Aug 28;10(8):e0135926. doi: 10.1371/journal.pone.0135926 (PMC4552654; doi:10.1371/journal.pone.0135926)
Supplement: S1 Table — Features 1–4 are specific to runs “coarse” and “coarseCon”, while features 5–24 are the forest types defined in Material & Methods. (DOCX) [file pone.0135926.s004.docx]

| **Run** | **ID** | **Tree species group** | **Soil fertility type** | **Weight** |
| --- | --- | --- | --- | --- |
| ”coarse”  “coarseCon” | 1 | Birch | NA | 1.3 |
|  | 2 | Spruce | NA | 1 |
|  | 3 | Other deciduous | NA | 2.6 |
|  | 4 | Pine | NA | 1 |
| “coarseClass”  “coarseClassCon”  “detailClass”  “detailClassCon” | 5 | Birch | Herb-rich | 4 |
|  | 6 | Birch | Herb-rich like | 2 |
|  | 7 | Birch | Mesic | 1 |
|  | 8 | Birch | Semi-xeric | 1 |
|  | 9 | Birch | Xeric | 1.5 |
|  | 10 | Spruce | Herb-rich | 3 |
|  | 11 | Spruce | Herb-rich like | 1.5 |
|  | 12 | Spruce | Mesic | 1 |
|  | 13 | Spruce | Semi-xeric | 1 |
|  | 14 | Spruce | Xeric | 1 |
|  | 15 | Other deciduous | Herb-rich | 7 |
|  | 16 | Other deciduous | Herb-rich like | 4 |
|  | 17 | Other deciduous | Mesic | 3 |
|  | 18 | Other deciduous | Semi-xeric | 2 |
|  | 19 | Other deciduous | Xeric | 2.5 |
|  | 20 | Pine | Herb-rich | 3 |
|  | 21 | Pine | Herb-rich like | 1 |
|  | 22 | Pine | Mesic | 1 |
|  | 23 | Pine | Semi-xeric | 1 |
|  | 24 | Pine | Xeric | 1 |
