# Supplementary material for: What Data to Use for Forest Conservation Planning? A Comparison of Coarse Open and Detailed Proprietary Forest Inventory Data in Finland
Source: PLoS One. 2015 Aug 28;10(8):e0135926. doi: 10.1371/journal.pone.0135926 (PMC4552654; doi:10.1371/journal.pone.0135926)
Supplement: S3 Table — The numbers on the header row and column correspond to the feature IDs in S1 Table. Note that the matrix is asymmetrical, i.e. the direction of the connectivity effect matters. Columns represent the forest types afflicting the connectivity effect, rows the forest types receiving the connectivity effect. (DOCX) [file pone.0135926.s006.docx]

|  | **5** | **6** | **7** | **8** | **9** | **10** | **11** | **12** | **13** | **14** | **15** | **16** | **17** | **18** | **19** | **20** | **21** | **22** | **23** | **24** |
| --- | --- | --- | --- | --- | --- | --- | --- | --- | --- | --- | --- | --- | --- | --- | --- | --- | --- | --- | --- | --- |
| **5** | 1.00 | 0.95 | 0.85 | 0.75 | 0.60 | 0.80 | 0.75 | 0.65 | 0.55 | 0.40 | 0.95 | 0.90 | 0.80 | 0.70 | 0.55 | 0.70 | 0.65 | 0.55 | 0.45 | 0.30 |
| **6** | 0.95 | 1.00 | 0.95 | 0.80 | 0.70 | 0.75 | 0.80 | 0.75 | 0.60 | 0.50 | 0.90 | 0.95 | 0.90 | 0.75 | 0.65 | 0.65 | 0.70 | 0.65 | 0.50 | 0.40 |
| **7** | 0.85 | 0.95 | 1.00 | 0.95 | 0.85 | 0.65 | 0.75 | 0.80 | 0.75 | 0.65 | 0.80 | 0.90 | 0.95 | 0.90 | 0.80 | 0.55 | 0.65 | 0.70 | 0.65 | 0.55 |
| **8** | 0.80 | 0.85 | 0.95 | 1.00 | 0.95 | 0.60 | 0.65 | 0.75 | 0.80 | 0.75 | 0.75 | 0.80 | 0.90 | 0.95 | 0.90 | 0.50 | 0.55 | 0.65 | 0.70 | 0.65 |
| **9** | 0.65 | 0.70 | 0.85 | 0.95 | 1.00 | 0.45 | 0.50 | 0.65 | 0.75 | 0.80 | 0.60 | 0.65 | 0.80 | 0.90 | 0.95 | 0.35 | 0.40 | 0.55 | 0.65 | 0.70 |
| **10** | 0.80 | 0.75 | 0.65 | 0.55 | 0.40 | 1.00 | 0.95 | 0.85 | 0.75 | 0.60 | 0.79 | 0.74 | 0.64 | 0.54 | 0.39 | 0.85 | 0.80 | 0.70 | 0.60 | 0.45 |
| **11** | 0.75 | 0.80 | 0.75 | 0.60 | 0.50 | 0.95 | 1.00 | 0.95 | 0.80 | 0.70 | 0.74 | 0.79 | 0.74 | 0.59 | 0.49 | 0.80 | 0.85 | 0.80 | 0.65 | 0.55 |
| **12** | 0.65 | 0.75 | 0.80 | 0.75 | 0.65 | 0.85 | 0.95 | 1.00 | 0.95 | 0.85 | 0.64 | 0.74 | 0.79 | 0.74 | 0.64 | 0.70 | 0.80 | 0.85 | 0.80 | 0.70 |
| **13** | 0.60 | 0.65 | 0.75 | 0.80 | 0.75 | 0.80 | 0.85 | 0.95 | 1.00 | 0.95 | 0.59 | 0.64 | 0.74 | 0.79 | 0.74 | 0.65 | 0.70 | 0.80 | 0.85 | 0.80 |
| **14** | 0.45 | 0.50 | 0.65 | 0.75 | 0.80 | 0.65 | 0.70 | 0.85 | 0.95 | 1.00 | 0.44 | 0.49 | 0.64 | 0.74 | 0.79 | 0.50 | 0.55 | 0.70 | 0.80 | 0.85 |
| **15** | 0.90 | 0.85 | 0.75 | 0.65 | 0.50 | 0.79 | 0.74 | 0.64 | 0.54 | 0.39 | 1.00 | 0.95 | 0.85 | 0.75 | 0.60 | 0.65 | 0.60 | 0.50 | 0.40 | 0.25 |
| **16** | 0.85 | 0.90 | 0.85 | 0.70 | 0.60 | 0.74 | 0.79 | 0.74 | 0.59 | 0.49 | 0.95 | 1.00 | 0.95 | 0.80 | 0.70 | 0.60 | 0.65 | 0.60 | 0.45 | 0.35 |
| **17** | 0.75 | 0.85 | 0.90 | 0.85 | 0.75 | 0.64 | 0.74 | 0.79 | 0.74 | 0.64 | 0.85 | 0.95 | 1.00 | 0.95 | 0.85 | 0.50 | 0.60 | 0.65 | 0.60 | 0.50 |
| **18** | 0.70 | 0.75 | 0.85 | 0.90 | 0.85 | 0.59 | 0.64 | 0.74 | 0.79 | 0.74 | 0.80 | 0.85 | 0.95 | 1.00 | 0.95 | 0.45 | 0.50 | 0.60 | 0.65 | 0.60 |
| **19** | 0.55 | 0.60 | 0.75 | 0.85 | 0.90 | 0.44 | 0.49 | 0.64 | 0.74 | 0.79 | 0.65 | 0.70 | 0.85 | 0.95 | 1.00 | 0.30 | 0.35 | 0.50 | 0.60 | 0.65 |
| **20** | 0.70 | 0.65 | 0.55 | 0.45 | 0.30 | 0.85 | 0.80 | 0.70 | 0.60 | 0.45 | 0.70 | 0.65 | 0.55 | 0.45 | 0.30 | 1.00 | 0.95 | 0.85 | 0.75 | 0.60 |
| **21** | 0.65 | 0.70 | 0.65 | 0.50 | 0.40 | 0.80 | 0.85 | 0.80 | 0.65 | 0.55 | 0.65 | 0.70 | 0.65 | 0.50 | 0.40 | 0.95 | 1.00 | 0.95 | 0.80 | 0.70 |
| **22** | 0.55 | 0.65 | 0.70 | 0.65 | 0.55 | 0.70 | 0.80 | 0.85 | 0.80 | 0.70 | 0.55 | 0.65 | 0.70 | 0.65 | 0.55 | 0.85 | 0.95 | 1.00 | 0.95 | 0.85 |
| **23** | 0.50 | 0.55 | 0.65 | 0.70 | 0.65 | 0.65 | 0.70 | 0.80 | 0.85 | 0.80 | 0.50 | 0.55 | 0.65 | 0.70 | 0.65 | 0.80 | 0.85 | 0.95 | 1.00 | 0.95 |
| **24** | 0.35 | 0.40 | 0.55 | 0.65 | 0.70 | 0.50 | 0.55 | 0.70 | 0.80 | 0.85 | 0.35 | 0.40 | 0.55 | 0.65 | 0.70 | 0.65 | 0.70 | 0.85 | 0.95 | 1.00 |
